# Modelling the Functions of Polo-Like Kinases in Mice and Their Applications as Cancer Targets with a Special Focus on Ovarian Cancer

**DOI:** 10.3390/cells10051176

**Published:** 2021-05-12

**Authors:** Monika Kressin, Daniela Fietz, Sven Becker, Klaus Strebhardt

**Affiliations:** 1 Institute for Veterinary Anatomy, Histology and Embryology, Justus Liebig University Giessen, 35392 Giessen, Germany; Daniela.Fietz@vetmed.uni-giessen.de; 2 Department of Gynecology, Goethe-University, 60590 Frankfurt, Germany; sven.becker@kgu.de (S.B.); strebhardt@em.uni-frankfurt.de (K.S.); 3 German Cancer Consortium (DKTK), German Cancer Research Center, Partner Site Frankfurt am Main, 60590 Frankfurt, Germany

**Keywords:** polo-like kinases, oncogenesis, cancer treatment, ovarian cancer, mouse models

## Abstract

Polo-like kinases (PLKs) belong to a five-membered family of highly conserved serine/threonine kinases (PLK1-5) that play differentiated and essential roles as key mitotic kinases and cell cycle regulators and with this in proliferation and cellular growth. Besides, evidence is accumulating for complex and vital non-mitotic functions of PLKs. Dysregulation of PLKs is widely associated with tumorigenesis and by this, PLKs have gained increasing significance as attractive targets in cancer with diagnostic, prognostic and therapeutic potential. PLK1 has proved to have strong clinical relevance as it was found to be over-expressed in different cancer types and linked to poor patient prognosis. Targeting the diverse functions of PLKs (tumor suppressor, oncogenic) are currently at the center of numerous investigations in particular with the inhibition of PLK1 and PLK4, respectively in multiple cancer trials. Functions of PLKs and the effects of their inhibition have been extensively studied in cancer cell culture models but information is rare on how these drugs affect benign tissues and organs. As a step further towards clinical application as cancer targets, mouse models therefore play a central role. Modelling PLK function in animal models, e.g., by gene disruption or by treatment with small molecule PLK inhibitors offers promising possibilities to unveil the biological significance of PLKs in cancer maintenance and progression and give important information on PLKs’ applicability as cancer targets. In this review we aim at summarizing the approaches of modelling PLK function in mice so far with a special glimpse on the significance of PLKs in ovarian cancer and of orthotopic cancer models used in this fatal malignancy.

## 1. Polo-Like Kinases and Their Physiological Functions

**Figure 1:**
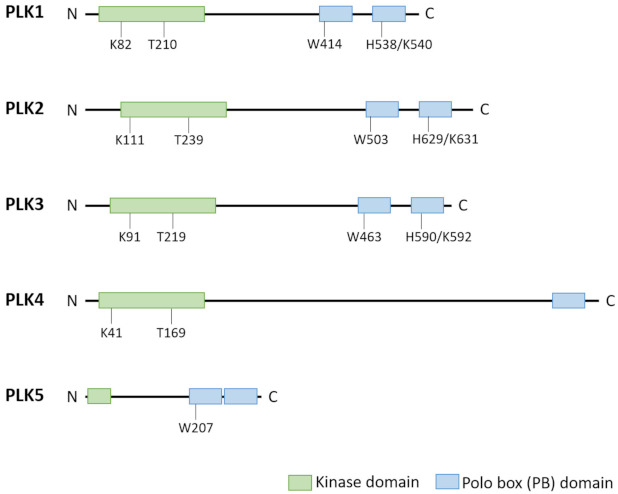
Structural differences between PLK family members PLK1–PLK5 [16]. PLK family members show a similar structure except PLK5. PLK1–PLK4 contain a highly conserved kinase domain at the *N*-terminal end and a non-catalytic polo box domain (PBD) at the *C*-terminus. The PBD is formed by three polo-boxes (PLK4) or two polo box motifs. These PBs are involved in substrate binding and regulation of kinase activity. Key residues of the kinase domain (acceptor lysine and T-loop threonine) and the PBs for substrate recognition are indicated. PLK5 has lost its catalytic activity in humans and expresses only a small portion of the kinase domain along with the PBD, the second PB has lost the conserved key residue involved in phosphosubstrate binding.

The founding name *polo* deduces from the effect of the gene-knockout first described in *Drosophila melanogaster* in form of abnormal spindle poles during mitosis [1]. Its orthologue in vertebrates encodes the protein polo-like kinase 1 (*PLK1*) which proofed to act as a key regulator of the cell cycle [2]. PLK1 belongs to a family of serine/threonine protein kinases, comprising five members PLK1 to PLK5 (in order of identification) in higher eukaryotes, among which PLK1 is far the most thoroughly studied and best characterized member (as reviewed by [3,4,5,6]). Family members (with the exception of PLK5) contain an ATP-binding catalytic serine/threonine kinase domain at the amino-terminus transferring a phosphate group to a multiplicity of cellular targets. Separated from the kinase domain by a linker region are, with the exception of PLK4, two polo-box motifs folding together and representing the non-catalytic regulatory domain in the carboxy-terminus. These motifs are known as the polo-box domain which is unique to the PLK family (for reviews see [7,8]). PLK4 is singular and the least representative family member with respect to its triple polo-box architecture including a cryptic dimeric one [9], for reviews see [10,11]. Catalytic as well as regulatory domains are evolutionary highly conserved. The polo-box domain recognizes and binds to ligands with phosphorylated serine/threonine motifs, thereby directing the enzyme to differential subcellular locations and target proteins as substrates and modulating the kinase activity [12,13,14], for reviews see [6,15]. PLK1, PLK2, and PLK3 share high sequence homology especially with respect to their kinase domain but to a lesser extent also to the polo-box domain. In contrast, PLK4 as well as PLK 5 reveal divergent structural properties (Figure 1).

**Figure 2:**
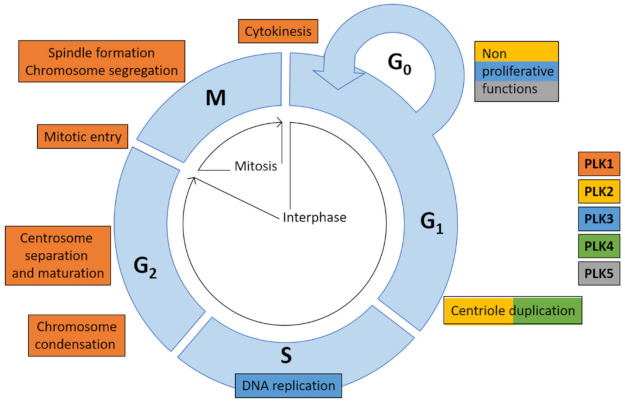
Functions of PLK family members in cell cycle [16]. PLK family members are essential for cell cycle processes, such as centriole duplication (PLK2 and PLK4), DNA replication (PLK3), chromosome condensation, centrosome separation and maturation, mitotic entry, spindle formation and chromosome segregation and cytokinesis (all PLK1). Besides cell cycle-related functions PLKs exert a multitude of vital cellular functions being part of complex signaling networks.

Family members are different in expression patterns within cells and tissues, subcellular localizations and functions and some exhibit a tissue-specific or -restricted expression. The PLK family, in particular PLK1 and PLK4, is best known for the orchestration of key events during mitosis (Figure 2) and plays important roles in cell cycle progression and regulation as well as in cell response to various types of stress (for reviews see [3,6,17,18]). Their differentially expressed members are key players in complex signaling networks controlling vital cellular functions [4,16,19,20].

PLK family members have partial-or non-overlapping substrates and each kinase serves specific functions in a wide variety of cellular processes. PLK1 and PLK4 share high expression in actively proliferating cells and tissues predominantly during S/G2 and M-phase contrary to PLK2, PLK3, and PLK5 (Figure 2).

*PLK1* (also known as PLK and STPK13) is highly expressed in embryonic as well as in adult cells and tissues with intense proliferation. It is essential for life as *Plk1*-deficient mice are embryonic lethal [21] although *Plk1* haplo-insufficient mice are viable [22]. *PLK1* is a pivotal gene for the regulation and control of cell cycle progression and is involved in almost every event of the mitotic cell division itself (for reviews see [3,5,6,8,17,23,24]). Accordingly, expression of *PLK1*/PLK1 is low during interphase and high during mitosis at both mRNA and protein level with a peak at G2/M phase transition. Expression levels remain high until mitotic exit [25]. Intracellular half-life is quite short with approx. 9 h reviewed by [26,27]. The expression is p53-dependent and follows the upregulation of the mitotic kinases Cyclin B1 and cyclin-dependent kinase 1 (CDK1) during cell division [28]. In addition, PLK1 is a central actor during meiosis [29,30] and reviewed by [20]. As a multi-functional mitotic kinase, PLK1 is localized to various subcellular locations depending on the cell cycle stage and it exerts stage-dependent specific functions throughout most of the cycle (for reviews see [6,31]). During interphase, PLK1 is associated with centrosomes as it is required for centrosomal maturation in G2 [32,33]. It participates in checkpoint recovery, the timing of mitotic entry, chromosome condensation and bipolar spindle formation [34,35,36]. Via stabilization of microtubule-kinetochore attachment [37,38,39,40], PLK1 ensures accurate chromosome alignment in the metaphase plate and orchestrates correct chromosomal segregation in anaphase [41,42]. Finally, PLK1 coordinates execution of cytokinesis and mitotic exit [43,44,45].

However, the multiplicity of substrates of PLK1 beyond mitosis (for reviews see [31,46,47]) suggests much more complex roles in cell cycle regulation and underlines vital interphase-related cellular functions far beyond the traditional mitotic ones which are controlling microtubules in the centrosomes, the spindle and the kinetochore. These non-mitotic functions include ciliogenesis, DNA replication, transcription and translation, stress signaling and cell responses to DNA damage as well as the regulation of tumor suppressor p53 activity and the targeting of the apoptotic signaling pathway [6,17,46,47,48,49,50,51]. PLK1 is engaged in autophagy-mediating pathways involving mechanistic target of Rapamycin (mTOR) as a key kinase promoting (cancer) cell growth [52,53,54].

*PLK2* (also known as serum inducible kinase SNK, hSNK or hPlk2) [55] is predominantly expressed in non-proliferating tissues and not associated with high mitotic rates. It is found in a rather tissue-specific manner compared to *PLK1* [19,56]. Attention has been drawn to participation in development, especially of the mammary gland epithelium [57,58] and to non-catalytic roles in the adult nervous system, where it is highly expressed and associated with the control of neuronal activity and synaptic function [59,60,61,62] repressing synaptic hyperactivity [63]. Mitosis-associated kinase activity is seen in centriole duplication, were PLK2 is localized during early G1 phase [64,65]. In addition, it plays a role in S-phase checkpoint [66]. Intracellular half-life is short (reviewed by [26]). PLK2 is involved in genotypic stress response and upregulated after DNA-damage, thereby participating in the activation of a p53-dependent G2 checkpoint and ensuring genomic stability [67].

PLK3 (also named cytokine inducible kinase CNK, FGF-inducible kinase FNK or proliferation-related kinase PRK) is a least explored family member with diverse functions found in a variety of tissues and organs (e.g., ovary, testis, skin, placenta, brain, lung, gastrointestinal mucosa, and hematopoietic tissues). *Plk3*-deficient mice develop rather normal and are fertile [68,69] which may, however, result from compensatory mechanisms. Its expression is cell cycle-regulated and peaks transiently during G1 phase. During mitosis, PLK3 is in close association with spindle poles and mitotic spindle [70]. The protein is very stable (reviewed by [26]). PLK3 is enriched at the plasmalemma and at Golgi membranes [71,72], reviewed by [73], possibly involved in the modulation of cellular adhesion and in intracellular trafficking [71]. Importantly, PLK3 participates in the Fas ligand-induced pathway of apoptosis [74], reviewed by [72]. Supposed roles for the progression of the cell cycle, namely entry into S phase are questioned [69,75] whereas PLK3 has been shown to participate in G2/M transition through interaction with cell division cycle Cdc25C protein phosphatase [76]. PLK3 has been reported to function in the mediation of cellular response including cell cycle checkpoint activation [77,78] after various forms of stress including hypoxic, DNA damage and osmotic stress [79,80], for reviews see [47,72]. Results from *Plk3*-knockout and its inactivation, however, argue against significant roles in cellular stress response [73]. The current data underline that PLK3 regulatory role in the cellular network is rather complex and diverse [72,81].

*PLK4* (also known as SAK, STK18 or MCCRP2) is highly expressed in actively dividing cells during development and in adult tissue as accounts for PLK1 [19,82]. *Plk4*-deficiency leads to embryonic death [83]. Contrary to *PLK1*, its expression starts in G1-phase and gradually increases to a peak in G2-phase with differential localization according to cell cycle stage [83]. The protein is quickly degraded (reviewed by [26]). PLK4 is essential for centriole biogenesis driving centriole duplication and mitotic progression [84,85,86,87], for reviews see [6,10,11,47,88,89]. Like PLK1, PLK4 acts as an integrative protein involved in the control of cell division. It localizes to the centrosome, kinetochore, cleavage furrow and midbody during the course of the cell cycle and participates in the maintenance of chromosomal stability [90,91]. PLK4 triggers mitosis via phosphorylation of Cdc25 [92]. PLK4 is required for cytokinesis. It is a microtubule-associated protein capable of promoting de novo centrosome formation [93]. Thus, PLK4 and PLK1 are key mitosis- and cell cycle orchestrating kinases taking over separate functions with both being tightly regulated. PLK4 substrates are not restricted to centriolar-associated networks but a multiplicity of potential target substrates point to participation in non-centriolar signaling networks [94]. PLK4 interacts with a variety of proteins including p53 and nuclear factor kappa B (NFҡB) affecting its expression, stability and activity (reviewed by [10,89]).

PLK5 (also known as PLK-5, PLK5P, and SgK384ps) is less explored but unique member of the family as it lacks typical kinase activity due to a truncated kinase domain [95]. *PLK5* expression is restricted to a few tissues. These are eye, male and female tissues and mainly brain, were it is implicated in neuron differentiation, namely axonal outgrowth, and neuronal activity [96], reviewed by [6]. PLK5 localizes to the nucleolus, is downregulated in proliferating cells and ectopically expressed in response to multiple stressors, esp. DNA damage or microtubule disruption, inducing G1 cell cycle arrest and apoptotic cell death [95], suggesting a cell-protective function.

## 2. PLKs and Tumor Development

Commonly, *PLK1* and *PLK4* have been considered as oncogenes whereas tumor-suppressive functions are attributed to *PLK2*, *PLK3*, and *PLK5*. With increasing knowledge, it becomes evident, however, that the participation and the mechanisms of action of the polo-like kinase family members in carcinogenesis are much more diverse and complex and far away from being fully understood. 

### 2.1. PLK1 Is a Dual Game Player in Tumorigenesis

The role of PLK1 in the context of tumorigenesis is complex and conflicting. On the one hand it is considered as an oncogene closely linked to human cancer development and on the other hand tumor suppressor functions are attributed to PLK1.

#### 2.1.1. Oncogenic Potential of PLK1

According to the critical regulatory role of PLK1 during mitosis, expression of *PLK1* is below a threshold of detection in normal tissues with low proliferation rates like heart, lung, or brain. Contrary, *PLK1* levels are high in actively proliferating cells and tissues like testis, bone marrow, spleen or thymus, during embryonic development and, of special interest, in tumor cells [2,97,98]. Dysregulation of *PLK1* is a hallmark of a multitude of cancers [99,100]. Since the first report of elevated *PLK1*-levels in human cancer compared to normal, non-transformed cells [97] and malignant cell transformation initiated by constitutive expression of *PLK1* [101], *PLK1* has been shown to be overexpressed in a multitude of malignancies (reviewed by [102]) such as lung cancer [103,104,105], esophageal and gastric cancer [106,107,108], oropharyngeal carcinomas [109,110], breast cancer [103,104,111,112], melanoma [113,114,115,116], various non-melanoma skin cancers [117], (colo)-rectal cancer [118,119,120,121], medulloblastoma [122], mesothelioma [123], glioma [124], neuroblastoma [125,126], endometrial cancer (reviewed by [127]), thyroid cancer [128], pancreatic cancer [129], prostate cancer [130,131], hepatoblastomas [132], hepatocellular carcinomas [133,134,135], non-Hodgkin lymphomas [136,137], bladder [138] and renal cancer [139], and ovarian cancer [2,140,141,142,143,144]; for detail see below). High levels of *PLK1* are widely linked to oncogenic transformation, cancer progression, invasiveness, high metastatic potential and, importantly, poor overall patient survival [7,17,26,102,104,105,106,108,109,110,111,112,113,120,125,127,130,132,134,135,139,144,145]. *PLK1* expression has been commonly considered as a key player in cancer development capable of serving as a tumorigenic biomarker and poor-prognostic predictor (reviewed by [127]). This commonly led to the consideration of *PLK1* as a classical oncogene.

Nevertheless, the precise role of *PLK1* in the context of carcinogenesis—driving transformation by itself or solely contributing to an already initiated or established transformation—is under debate. In the light of highly proliferating tumor cells and the cell cycle-dependent expression of the mitotic kinase, high expression of *PLK1* may not be a cause but a consequence during tumorigenesis just reflecting high mitotic rate [146]. Concordant with the oncogenic conception, however, convincing evidence is accumulating that PLK1 is actively involved in the fatal events throughout tumorigenesis from the onset of oncogenic transformation, tumor growth, epithelial-mesenchymal transformation, tumor cell invasion and metastasis and, last but not least, PLK1 is supposed to participate in development of therapeutic resistance [46,146,147] and reviewed by [148]. PLK1 directly participates in oncogenic signaling and interacts with a variety of oncogenic pathways (for reviews see [102,147,149]). Important in this context is the complex and multifaceted intertwining relationship between PLK1 and several transcription factors. Among these is the p53 protein encoded by the key tumor suppressor gene *TP53* which is a critical target of PLK1 (reviewed by [150]). Loss or mutation of the genome-guarding *TP53* and impaired p53 function account for the majority of tumor cells [151], reviewed by [152] and without functional p53, downregulation of *PLK1* is impaired leading to elevated PLK1 levels [102,111,149]. Overexpression of *PLK1* accelerates the cell cycle, enables cells to override cell cycle checkpoints leading to mitotic defects and favoring chromosomal instability and aneuploidy [48,99,100]. In a PLK1-p53 negative feedback-loop, overexpression of *PLK1* is, in turn, linked to inhibition of p53 function promoting tumorigenic status [153]. *PLK1* knockdown via RNA interference leads to *p53* upregulation in an ovarian cancer xenograft model [154]. A positive feedback-loop exists between PLK1 and the oncogenic transcription factor MYC [153]. Another tumorigenic molecular mechanism of PLK1 is promoting the inactivation of tumor suppressors like the phosphatase and tensin homologue *PTEN* [149,155]. Disruption of *PTEN* frequently occurs in cancer (reviewed by [49]). *PLK1* expression may even critically regulate *PTEN* expression acting bi-functionally [156]. Besides TP53 and PTEN, PLK1 interacts with several other tumor suppressors and oncogenes like Forkhead box protein M1 FOXM1 and members of the MYC family (for reviews see [102,147]). Another oncogenic mechanism of PLK1 is targeting metabolic pathways in order to establish a tumor-adapted and growth-favoring cellular metabolism [155,157,158]. Last not least, *PLK1* overexpression is critically involved in epithelial-mesenchymal transition, whereby well-differentiated epithelial cells acquire characteristics of rather poorly differentiated mesenchymal cells as a prerequisite for cancer cell motility, invasiveness, dissemination and metastasis. Epithelial-mesenchymal transition is one of the key processes in aggressive tumorigenesis [159,160]. This fatal transformation implies profound and dynamic alteration and re-organization of the actin-myosin and intermediate filament cytoskeleton including cell-cell and cell-matrix junctions [161,162]. *PLK1* overexpression induces and is linked to these epithelial-mesenchymal transition-associated key events via activation of defined and cancer-type specific signaling cascades [163,164,165]. These may include AKT, FOXM1- and MAPK-dependent pathways.

#### 2.1.2. Tumor Suppressor Potential of PLK1

In contrast to the “pro-tumor” oncogenic potential of PLK1, there is strong evidence for PLK1 also acting as a tumor suppressor. Reduced PLK1 levels have been linked to tumorigenesis in *Plk1*-heterozygous mice [21], though in other PLK1-reduced settings a cancer-promoting effect of low and drastically reduced PLK1 could not be detected [22,98,166]. For some human malignancies such as breast cancer, elevated PLK1 levels are beneficial and associated with better prognosis [111] though reduced levels have also been reported to be exacerbating in this cancer entity [104]. With respect to breast cancer development, a tumor suppressor function of PLK1 has been confirmed in a *Plk1* gain of function mouse model using an inducible knock-in-setting [100]. For colorectal cancer, overexpression of *PLK1* is indicative of a favorable prognosis [167] with the same effect being revealed for colorectal tumors in a *Plk1*-inducible loss of function mouse model [99]. 

A possible explanation for these contradictory functions of PLK1 could be differences in the genetic background of cancer cells and tumor tissue. In colon cancer with chromosomal instability due to a non-sense APC mutation, elevated PLK1 levels have tumor-suppressive potential and increase the survival of patients [99]. A pan-cancer mRNA sequencing data analysis correlates *PLK1* expression levels with clinical parameter (patient overall survival) and reveals striking differentiated tumor-specific relations. Elevated PLK1 levels are associated both with good prognosis (e.g., in rectal adenocarcinoma, lung squamous cell carcinoma, and thymoma) and bad prognosis (e.g., in lung adenocarcinoma, bladder carcinoma, and kidney clear cell carcinoma) or are obviously not correlated (e.g., in stomach adenocarcinoma, cervical carcinoma, and ovarian cancer) depending on cancer type (reviewed by [149]). This diverging role of PLK1 reflects best the role of tumor heterogeneity and highly dynamic tumor change during growth and progression as well as during treatment. The role of PLK1 in ovarian cancer is a special interest of this review and will be discussed further below.

### 2.2. PLK2 and Tumorigenesis

The role of PLK2 in tumorigenesis is rather complex (reviewed by [47]). *PLK2* is downregulated in several cancers and therefore has been considered as a tumor suppressor [67]. This accounts for glioblastoma [168], hepatocellular carcinoma [169], acute myeloid leukemia [170], and for B-cell malignancies [171] where *PLK2* is epigenetically silenced, as well as for cervical cancer where PLK2 exhibits anti-proliferative and apoptosis-promoting effects [172]. This accounts for gastric cancer, too, according to siRNA-mediated *PLK2* knockdown [173]. In breast cancer, reduced *PLK2* expression is linked to adverse prognosis [174]. In colorectal adenocarcinomas, *PLK2* expression is completely or partially lost [175].

*PLK2* gene is targeted by p53 and it is induced after genotoxic stress in vivo [67]. In view of the frequent disruption and inhibition of the genome-guarding p53 pathway in a multitude of cancers (reviewed by [176]) tumor suppressor functions of PLK2 are impaired, too. In glioblastoma, PLK2 is negatively correlated with the central notch signaling pathway [168]. Downregulation of *PLK2* promotes tumor aggressiveness and chemo resistance via activation of notch axis and *PLK2* overexpression reduces malignant behavior both in vitro and in vivo.

The role of PLK2 in tumorigenesis is, however, conflicting. In colorectal carcinomas, PLK2 exerts tumor growth-promoting and apoptosis-inhibiting effects [167,175,177,178] as is the case for several other tumors like lung cancer, cholangiocarcinoma, osteosarcoma, and head and neck carcinoma [66,155,179,180,181,182]. An oncogenic function is also suggested for pancreatic cancer according to siRNA-induced *PLK2* silencing [183].

PLK2 has been demonstrated to mediate hedgehog survival signaling [180]. For certain cancers, as it is the case for cholangiocarcinoma, hedgehog signaling pathway is essential conferring apoptotic resistance. In colorectal cancer, PLK2 targets the Fbxw7/Cyclin E pathway promoting G1 transition to S phase of the cell cycle. High expression of *PLK2* correlates with high expression of Cyclin E promoting tumorigenesis [177]. In the same malignancy, the transcription factor forkhead box family member FOXD1 promotes *PLK2* expression on mRNA and protein levels resulting in increased proliferation and suppressed apoptosis of cancer cells [178].

Besides tumor-associated roles, PLK2 is critically involved in the pathogenesis of Alzheimer’s disease as elevated levels promote production of amyloid beta plaques in a mouse model [184] and also in synaptic plasticity [59]. The role of PLK2 in ovarian cancer is of special interest for this review and will be discussed further below.

### 2.3. PLK3 and Tumorigenesis

PLK3 is critically involved in oncogenesis (reviewed by [72]) and dysregulated expression of *PLK3* is found in a variety of tumors.

Expression of *PLK3* is reduced in various cancers like lung [71,185,186], hepatocellular carcinoma [169], head and neck squamous cell carcinoma [187], anal squamous cell carcinoma [74] as well as melanoma, liver, kidney, stomach, rectum [81], colon tumors [188], bladder, and uterus cancer [186]. Therefore, PLK3 is widely considered as a tumor suppressive kinase [26,68]. Supportive for this conception is the apparently more frequent development of spontaneous tumors in aged *Plk3*-deficient mice [68] although the correlation between *PLK3*-deficiency and higher tumor incidence is questioned [69].

Elevated expression of *PLK3*, however, is reported to account for breast cancer [104], prostate cancer [189], and hepatoblastoma [186] and is associated with poor prognostic impact. In colorectal cancer, overexpression of *PLK3* has been shown to correlate with unfavorable prognosis based on gene expression analysis [167]. Increased *PLK3* expression corresponds with better disease-related outcome in treated cervical carcinoma and low *PLK3* expression is associated with therapeutic resistance and increased metastasis based on immunohistochemical and genomic analyses [190] making PLK3 and its substrate pT273 Caspase 8 a valid prognostic marker.

The reasons for these differential cancer type-related and possibly development-associated expression patterns of *PLK3* remain elusive. PLK3 has been found to be a critical player in cellular hypoxic responses, which contribute to tumorigenesis and tumor progression [191,192,193]. The intimate involvement of PLK3 in various stress responses including DNA damage response implies cell cycle arrest and induction of apoptosis [72,79]. In this context PLK3, mediates p53-dependent and p53-independent pathways [72,77,194]. PLK3 functions in a pro-apoptotic response after induction by transcription factor NFҡB [195].

*Plk3*-deficient mice frequently develop tumors which are intensely vascularized [68]. This points to an essential role of PLK3 as a mediator of tumor angiogenesis, which is a prerequisite for tumor growth [192]. PLK3 acts via phosphorylating hypoxia inducible factor HIF-1α as a key protein in the angiogenic pathway [81]. The role of PLK3 in ovarian cancer is of special interest for this review and will be discussed further below.

### 2.4. PLK4 and Tumorigenesis

As accounts for *PLK1*, *PLK4* is overexpressed in a variety of solid tumors and hematologic malignancies considered to act as an oncogene (for reviews see [11,89]). High levels of PLK4 correlate with tumor growth, aggressive progression and treatment resistance representing a poor-prognostic marker. This accounts for a variety of malignancies including several epithelial cancer types (reviewed by [11,89]) such as breast cancer [196,197,198,199], lung cancer [200,201], prostate cancer [202], and gastric cancer [203,204], but also for neuroblastoma [205], glioblastoma [206], and acute leukemia [207,208]. In skin epidermis, overexpression of *PLK4* leads to hyperplasia [209] and promotes—in association with p53 deficiency—tumorigenesis causing hyperproliferation and abnormal differentiation of basal keratinocytes and melanocytes [209,210]. Additionally, melanoma overexpress *PLK4* [211]. For colorectal cancer, data concerning effects of *PLK4* overexpression are conflicting as it has been shown to be related to a favorable prognosis [167] as well as to an adverse disease development and unfavorable prognosis [212,213,214]. In hepatocellular carcinoma, downregulation of *PLK4* is reported to be linked to larger tumor size and poor prognosis [40,169] as it has been observed for overexpressed *PLK4* [213]. These contradictory correlations point to PLK4 possibly acting as a tumor promoter as well as a tumor suppressor depending on cancer biology.

In view of the pivotal role of PLK4 in centriole duplication, mitotic progression, and cytokinesis, dysregulated *PLK4* promotes oncogenic mitotic errors and genomic inconstancy. *Plk4^+/−^* heterozygous mice are viable but frequently develop spontaneous lung and liver tumors compared with *Plk4*^+/+^ homozygous mice [215]. Overexpressed *PLK4* induces centrosomal amplification and disturbs cytokinesis while downregulated expression of *PLK4* disturbs formation of the spindle apparatus [84,91,209,210]. Both lead to chromosomal instability, which implies a high rate of chromosome abnormalities (reviewed by [216]), aneuploidy and aberrant cell proliferation, hallmarks of human cancer cells [203,217,218]. Chromosomal instability and aneuploidy is negatively correlated with aggressiveness and metastasis of cancer, with adaptive resistance to therapies and with bad prognosis [219,220,221]. Amplificated centrosomes are sufficient to induce aneuploidy and to promote spontaneous tumorigenesis possibly involving downregulation of the p53 pathway [222]. Overexpressed *PLK4* has been shown to activate the ataxia telangiectasia and Rad3-related (ATR)-checkpoint kinase 1 (CHEK1) signaling pathway, which is critically involved in DNA damage response and genomic stability [213]. This pathway is supposed having a tumor-promoting function in several cancers. In addition, PLK4 induces epithelial-mesenchymal transition (for reviews see [11,89], which is one of the key processes in aggressive tumorigenesis as depicted for oncogenic function of overexpressed *PLK1* (see above). Cancer cell motility and invasiveness imply dynamic cytoskeletal re-organization and junctional cell-cell as well as cell-matrix (re)modelling [161,162]. *PLK4* overexpression affects normal intercellular adhesion and promotes invasiveness through activation of oncogenic signaling pathways [223]. PLK4 drives epithelial-mesenchymal transition of cancer cells, cancer cell motility and migration. PLK4 acts via the phosphatidylinositol 3′ kinase PI3K/AKT pathway and via the actin-related protein ARP 2/3 complex mediating actin cytoskeletal rearrangement and aiming at cadherin-conveyed cell adhesion [205,224,225,226]. PLK4 is also part of WNT signaling pathways involved in the regulation of cancer cell motility [227] and invasiveness [214]. In addition, PLK4 may be responsible for development of drug resistance to taxane-based neoadjuvant chemotherapy through the induction of tubulin-mutations [199]. The role of PLK4 in ovarian cancer is of special interest for this review and will be discussed further below.

### 2.5. PLK5 and Tumorigenesis

PLK5 acts as a tumor suppressor in human brain cancer as it is silenced in glioblastoma [96]. A defined mutation of *PLK5* is specifically implicated in metastasis of human renal cell carcinoma [228].

## 3. Targeting PLKs in Cancer

In view of the multilayered significance of dysregulated PLKs in the context of tumorigenesis, family members are targeted in cancer treatment. This accounts especially for *PLK1* and *PLK4* as they are selectively overexpressed in a broad range of tumor cells and tissues compared to healthy ones (for reviews see [7,11,17,148,229]).

Since the first reports of *PLK1* downregulation with anti-sense oligonucleotides and small interfering RNA inducing growth inhibition in cancer cells [230,231,232] and in living animals [27,232] a plethora of studies have been performed focusing on PLK1 inhibitors suitable as anti-cancer drug [233,234,235,236,237] reviewed by [238,239,240]. Inhibiting PLK1 activity addresses actively dividing cells and leads to mitotic arrest at the G2-M transition, double-stranded DNA breaks and to induction of apoptosis [51,115,116,125,131,208,241,242,243]. Promising inhibitors targeting PLK1 have been investigated, approved in preclinical studies and some have reached clinical trials [240]. Remaining problems are limited therapeutic efficiency, cancer recurrence, development of resistance and side effects due to low specificity. It is important to realize that PLK1 inhibitors that are currently in the clinic might target to some extent rapidly dividing cell irrespective if they are malignant or healthy [150,242]. The third generation ATP-competitive PLK1 inhibitor, Onvansertib^®^, was tested in an array of 260 kinases, showed very high specificity toward PLK1 and could represent a major improvement compared to previous generations of ATP-competitive PLK1 inhibitors.

Three classes of PLK1 targeting drugs can be distinguished, namely ATP-competitors, polo-box domain competitors and RNA interference-based therapies. ATP-competitors address the kinase domain carrying the ATP-binding pocket. This domain is, however, shared by all family members (except PLK5) and by many other kinases. Therefore drugs targeting this pocket address also other kinase domains affecting, among others, the tumor suppressor functions of PLK2 and in particular of PLK3 leading to adverse side effects [244]. The most widely used (small molecule) inhibitors targeting the catalytic activity of PLK1 are BI2536 and BI6727 (Volasertib^®^) for reviews see [148,240]. Contrary, inhibitors targeting the unique polo-box domain of PLK1 reach higher specificity and minimize cross-reactivity with other kinases. The first designed polo-box domain-targeted inhibitor (Poloxin^®^) induces mitotic arrest [148,245,246,247] and inhibits tumor growth in vivo in a xenograft mouse model [248]. Several PLK1 polo-box domain competitors followed [148,207,240,249].

A promising and effective strategy for specific targeting of PLK1 without affecting other kinases, is the use of RNA interference (RNAi). RNAi drugs induce gene silencing by sequence-specific cleavage of targeted mRNA. The specificity as well as efficiency of this strategy has been demonstrated in a genetically engineered mouse model via a short hairpin RNA (shRNA) induced *Plk1* mRNA knockdown considerably reducing side effects [98,250]. Specific *PLK1*/PLK1 knockdown on both protein and mRNA level is achieved by short interfering Ribonucleic Neutrals (siRNNs) as pro-drugs which can be cleaved intracellularly into short interfering ribonucleic acids (siRNAs) as demonstrated in leukemia [251]. Effective and specific siRNA-*PLK1* inhibition could also be achieved in prostate cancer cells [131] and in acute myeloid leukemia [54]. Selective knockdown of *PLK1* mRNA is induced by siRNNs in acute lymphoblastic leukemia [208].

siRNA-silencing of *PLK2* gene promoted apoptosis during mitosis in various cancer cell lines (e.g., carcinoma of lung, cervix, breast and colon, and osteosarcoma) in the presence of spindle poisons like paclitaxel [67]. *PLK2*-silencing does not impair mitotic cycle progression in cells not exposed to microtubule poisons. Therefore, inhibition of PLK2 might be a therapeutic option for sensitizing Paclitaxel^®^-resistant tumors [67]. In cholangiocarcinoma, pharmacological inhibition of PLK2 via BI6727 and *PLK2* knockdown degrade the anti-apoptotic protein myeloid cell leukemia 1 (Mcl-1) which represents a survival factor in this malignancy, and lead to apoptosis and tumor suppression in vivo [180].

In view of the critical role of *PLK4* overexpression in a variety of malignancies, its inhibition is a therapeutic cancer strategy [10,11,91,229] for reviews see [11,89]. Several small molecules ATP competitive inhibitors have been developed, among which CFI-400945 is the most prominent. It affects centriole duplication and mitotic spindle formation, prevents cellular abscission and generates polyploid cells resulting in apoptotic death as demonstrated in breast cancer and colorectal cancer lines [91]. CFI-400945 inhibits lung cancer growth in mice [200]. Preclinical studies confirmed in vivo anti-tumor activity as it is the case for patient-derived xenograft (PDX) models, breast cancer, pancreatic cancer, and osteosarcoma [252,253]. Favorable results are obtained from a clinical study with various solid tumors [254]. Other inhibitors target the critical role of PLK4 in centriolar function and inhibit cell proliferation in melanoma cells [211], breast cancer [255], cervical carcinoma, and colon carcinoma [256,257] only to name a few.

## 4. Ovarian Cancer and PLKs

As normal ovarian tissue contains actively proliferating cell populations, *PLK1* is physiologically expressed at high levels [2,140,141,142,143]. The other members of the PLK family, especially *PLK2* and *PLK3*, are differentially expressed in periovulatory granulosa cells via hormonal induction [258].

Treatment of ovarian cancer representing the most lethal gynecological malignancy worldwide is a tremendous challenge [259]. Until now an effective screening strategy is missing to detect ovarian cancer at an early developmental stage, so it is often diagnosed at an advanced stage with bad prognosis due to aggressive metastatic progression, relapse after surgery and development of therapeutic resistance. Ovarian cancer comprises several divergent subtypes according to histological characteristics, molecular features, origin of cells (ovarian or extra-ovarian), risk factors, clinical features, and therapeutic response (for reviews see [260,261,262,263]). The most common subtype are epithelial ovarian carcinomas (about 90%) including the minor frequent subtypes mucinous, endometroid, clear cell subtype and the so-called serous ovarian carcinoma (about 75%) with a low-grade and high-grade variant of all serous ovarian carcinomas (about 70%). These various subtypes of ovarian cancer bear a multitude of significant molecular aberrations and genomic changes and are genetically marked heterogeneous as revealed by integrated genomic analysis based on the Cancer Genome Atlas [264] and other studies [262,265,266,267]. The specific genotype critically determines the response to therapeutic treatment [268,269].

### 4.1. PLK1 in Ovarian Cancer

As accounts for a multitude of other cancers, dysregulation of *PLK1* is linked to ovarian cancer. High expression of *PLK1* is reported to be associated with histological grade and clinical stage [143]. Elevated levels of *PLK1*/PLK1 mRNA and protein account for ovarian cancer cell lines and tissue and promote growth and migration of cancer cells and diminish apoptosis [270]. Overexpression of *PLK1* correlates positively with mitotic activity and is a prognostic factor in ovarian carcinoma linked to worse patient prognosis as judged from immunohistochemistry of several malignant epithelial subtypes [130]. This correlation is confirmed in a *PLK1* knockdown xenograft model pointing to PLK1 as an independent prognostic factor [271]. Highly expressed *PLK1* promotes proliferation and migration of cultured ovarian cancer cells [272]. In a large patient cohort with early-stage ovarian cancer, high *PLK1* expression correlates with bad prognosis based on immunohistochemistry [273]. Importantly, pharmacological induction of strong mitotic arrest via PLK1 inhibition and microtubule targeting followed by blocking mitotic exit activates apoptosis, prevents endoduplication and reduces chromosomal instability in an ovarian cancer cell line culture system [273]. Indirect evidence for the tumor-promoting effect of overexpressed *PLK1* in (relapsed) ovarian cancer comes from the induction of synthetic lethality in a patient-derived ovarian cancer cell culture [274] and from the clinically approved antitumor effect of the PLK1 inhibitor B16727 (Volasertib^®^) [275]. In a pan-cancer analysis using mRNA quantitative sequencing data, however, differing correlations between *PLK1* upregulation and overall survival of patients suffering from different cancer entities have been shown with *PLK1* overexpression not influencing overall survival for ovarian cancer (reviewed by [149]). In the mucinous subtype of ovarian carcinoma, downregulation of *PLK1* with siRNA as well as pharmacological PKL1 inhibition (Volasertib^®^ and Onvansertib^®^, both highly selective ATP-competitive PLK1 inhibitors) [276] in a xenograft model interferes with cell proliferation inducing mitotic arrest at G2/M phase leading to endoduplication as well as apoptosis [277]. The complex diversity of PLK1′s role in ovarian cancer becomes obvious in light of PLK1 serving as predictive marker for better prognosis with regard to the progression-free survival in a stage-dependent manner: PLK1 functions as a positive predictor in early-stage of a low-grade serous subtype but not in late-stage based on mRNA and protein expression [144]. Transcriptomic analysis of serous ovarian carcinomas reveals PLK-signaling events and PLK-dependent differentially expressed genes to be important in tumorigenesis and cancer progression [278].

Aurora borealis (*BORA*) is highly expressed in aggressive ovarian cancer and exerts its oncogenic role via activation of *PLK1* in vitro and in vivo [279]. BORA activates the key mitotic PLK1 function as a prerequisite for mitotic entry and G2/M checkpoint recovery and high BORA correlates with increased cancer cell proliferation and high grade of chromosomal instability [279]. One example of the complex network integration of PLKs is the interplay with microRNA (miRNA). miRNAs are short-sequence and not protein-encoding RNAs specifically binding to target mRNAs leading to their degradation or to inhibition of translation, thus post-transcriptionally regulating gene expression. miRNAs play important anti-oncogenic as well as oncogenic roles in the pathogenesis of ovarian cancer (for reviews see [280,281]). MiR-545 directly targets *PLK1* mRNA and inhibits *PLK1* expression in ovarian cancer thereby acting as a tumor suppressor as demonstrated in vitro and in vivo in a xenograft mouse model [270]. MiR-545 functions as a tumor suppressor and is lowly expressed in epithelial ovarian tissue whereas overexpression leads to inhibition of growth and increase in apoptosis [282].

### 4.2. PLK2 in Ovarian Cancer

*PLK2* downregulation is linked to ovarian tumorigenesis and drug resistance. In a *PLK2* knock-in and -knockdown model using primary cell culture, it could be demonstrated that therapeutic drug resistance is associated with transcriptional silencing of the kinase [283]. This was confirmed in a follow-up study [284] and is in accordance with significant downregulation of the *Plk2* gene identified in chemo resistant ovarian cancer via oligonucleotid microarrays [285]. Critical involvement of upregulated *PLK2* in resistance development is underlined by a transcriptome monitoring in isogenic ovarian cancer cells with gradually changing resistance [286]. 

### 4.3. PLK3 in Ovarian Cancer 

Contrary to many other cancers (see above), *PLK3* seems to act as an oncogene in the ovary. In different types of ovarian carcinoma, *PLK3* has been found to be overexpressed and to correlate with mitotic activity without being used, however, as a prognostic factor [130]. 

### 4.4. PLK4 in Ovarian Cancer

PLK4 seems to promote ovarian cancer. High expression of *PLK4*/PLK1 on mRNA and protein level is linked to an advanced pathological stage in epithelial ovarian cancer and *PLK4*-transfected ovarian cell lines show accelerated proliferation [287]. PLK4 is considered to serve as a biomarker and poor prognostic predictor in advanced stage of disease as well as therapeutic target.

### 4.5. PLK 5 in Ovarian Cancer

Allele-loss on *PLK5*-carrying chromosome locus is associated with a 50% increased risk of sporadic ovarian tumors among other neoplasia [288], suggesting a tumor-suppressive function of PLK5 in the ovary.

## 5. Targeting PLKs in Ovarian Cancer

Targeting PLKs in ovarian cancer bears diagnostic and prognostic potential as well as powerful therapeutic options. Late diagnosis of ovarian cancer at an advanced stage of disease is one cause of the high mortality rate. An important strategical goal must be the identification of markers suitable to detect ovarian cancer at early stage. PLK-dependent differentially expressed genes may serve as attractive biomarkers with prognostic value for early detection [278].

In order to effectively address tumor-specificity, a better staging of ovarian cancer including molecular markers for patient stratification is needed [289]. This is necessary in order to choose the appropriate treatment and to optimize that is personalize, therapeutic regime. Besides Ki67, PLK1 is a suitable stage-dependent marker. At least in a subtype of serous ovarian cancer this could be demonstrated on the level of mRNA expression [144]. The introduction of DNA microarray technique for determination of PLK levels in clinical use might be of strategical benefit.

The standard therapy for advanced disease (as most women present) is based on debulking surgery and chemotherapy with platinum derivatives (cisplatin, carboplatin) and taxanes, esp. Paclitaxel^®^ [290]. The former bind to DNA and inhibit DNA replication and transcription thus terminating cancer cell growth, the latter hyperstabilize microtubules perturbing mitotic spindle formation and correct chromosome segregation, which activates the mitotic spindle assembly checkpoint and prolongs mitotic arrest or inhibits mitosis. After initial responsiveness to chemotherapy most patients suffer from cancer recurrence with enhanced metastatic aggressiveness, acquired drug resistance, poor prognosis and ultimate death [291]. The reasons for this fatal course are complex and under intense examination. An important key to understanding is the marked inter- and intra-tumor heterogeneity of ovarian cancer bearing a multiplicity of mutations [264] and transcriptional silencing of many genes [292] affecting numerous key signaling pathways [293,294,295,296]. A very high degree of genome instability is characteristic, too [297]. Ideally, chemotherapy leads to death of these cancer cells. Affected cells, however, may not die via apoptosis but escape via mitotic slipping and continue cycling [298,299]. This means survival of cancer cells with an abnormal aneuploid genome and a high grade of chromosomal instability [300] driving fatal relapse with pronounced aggressiveness and metastasis. In particular, surviving ovarian cancer stem cells are thought to harbor malignancy and drug resistance [301]. In view of noteworthy populations of cancer cells not being killed by standard chemotherapy it is an urgent necessity as well as a tremendous challenge to optimize specified targeting. One strategy is addressing ovarian cancer-specific signaling pathways [293,294,301]. This essential knowledge may be beneficial in adjusting and personalizing therapy. A very promising strategy is a drug treatment regime including PLK1-inhibition. A therapeutic option is, among others, Volasertib^®^ (BI 6727), an ATP analogue designed as a PLK1-inhibitor targeting and inhibiting the kinase domain. Anticancer activity of Volasertib^®^ has been shown in patients with platinum-resistant or -refractory ovarian cancer [275]. Volasertib^®^ in a dual combination with the standard microtubule-targeting taxane, paclitaxel, induces synthetic lethality in different ovarian cancer cell lines including patient-derived ones [274]. This approach led to a significant increase in polyploid cells [273]. Pharmacological inhibition of PLK1 by BI2536 or Volasertib^®^ has been shown to induce mitotic arrest and slippage in a dose-dependent manner, that is with high concentration of PLK1 inhibitor [242]. BI 2536 was the first selective PLK1 inhibitor used in clinical trials for the treatment of advanced ovarian cancer [302]. Finally, Volasertib^®^ in combination with paclitaxel induces strong mitotic arrest in ovarian cancer cell lines and, when followed by pharmacological blocking of mitotic exit, prevents endoreduplication and reduces chromosomal instability [273]. This effective triple-combined strategy underlines the potency of PLK1 inhibition in the treatment of ovarian cancer. In mucinous ovarian carcinoma, specific downregulation of *PLK1* via siRNA and pharmacological inhibition via Volasertib^®^ or Onvansertib^®^ inhibits growth of cultured cell lines and induces apoptosis [277]. In transfer to an in vivo xenograft model best tumor growth inhibition is achieved via Onvansertib^®^ synergistically combined with paclitaxel.

Silencing of *PLK2* dramatically sensitizes an ovarian cancer cell line to anti-microtubule agents leading to apoptosis during mitosis [67].

A kinome-wide screening for modulators of the growth-inhibitory effect of cisplatin revealed that the inhibition of PLK3 sensitizes cultured ovarian cancer cells to chemotherapy [303].

In several ovarian cancer cell lines, inhibition of PLK4 kinase activity with the small-molecule inhibitor CFI-400945 inhibited proliferative activity and induced polyploidy [91]. Targeting PLK4 with the small molecule inhibitor YLZ-F5 prevents human ovarian cancer growth by inducing aneuploidy and promotes apoptosis via activation of caspases-3/9 and impairs cell migration [304].

## 6. Mouse Models in Ovarian Cancer Research

Important tools for the progress in ovarian cancer research are well-suited models. Besides classical methods using cell culture, mouse models based on xenografts and on genetic engineering are indispensable in ovarian cancer research [262,305]. They address the origin of ovarian cancer, genetic profiles and mutations, tumor growth and metastasis, effects of putative treatments as well as (therapeutic) response. 

**Figure 3:**
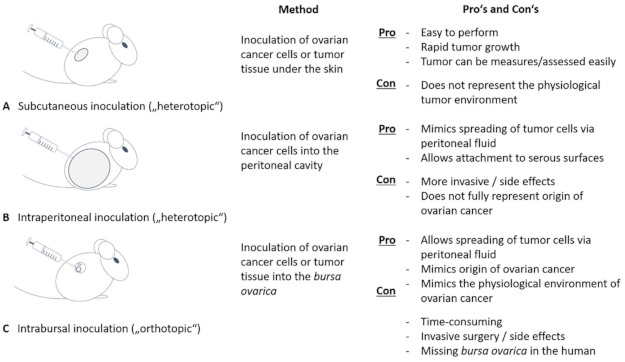
Mouse models in ovarian cancer research. Using mouse models have proved to be an important tool in ovarian cancer research. We differentiate between heterotopic mouse models, where ovarian cancer cells or cancer tissue is inoculated subcutaneously (**A**) or into the peritoneal cavity (**B**). Whereas a subcutaneous application is easy to perform and safe for the animal, it does not mimic the physiological tumor environment. In contrast, intraperitoneal application is able to imitate the spreading of tumor cells via peritoneal fluid and attachment to serous surfaces. As an orthotopic mouse model, intrabursal application is performed (**C**). The latter also imitates site of origin of ovarian cancer and mimics spreading and attaching of cancer cells, but is hampered by the fact that an ovarian pouch is missing in the woman.

Xenograft mouse models can be designed with patient-derived grafts or with well-defined cancer cell line grafts [306,307]. They are commonly used to gain knowledge about ovarian tumor biology and to screen therapeutic drug efficiency. Their value depends, besides above-mentioned types of graft, on the immune status of the host (immunodeficient or not) and on the method of engraftment. A common procedure is heterotopic engraftment, most commonly subcutaneous inoculation of ovarian cancer cells or tissue enabling rapid tumor growth easy in observation and measurement [277] (Figure 3).

However, subcutaneous tumor growth does not represent physiological environment. Interactions between non-malignant stromal cells surrounding and infiltrating tumors have been shown to critically determine tumor growth, spreading and response to therapy and are therefore decisive with respect to patient prognosis [154,308,309]. To get closer to a clinically relevant pathophysiological macro- and microenvironment, intraperitoneal tumor cell transplantation or an orthotopic model is preferable [310]. Tumor cells or a solid tumor are applicated intrabursally, i.e., into the bursa ovarica enclosing mouse ovary or directly onto the ovary, thereby reproducing the primary site of tumor growth and disease progression best [311]. Intrabursal as well as intraperitoneal inoculation allow tumor cells to spread throughout the peritoneal cavity and to attach to serosal surfaces thus representing a well-suited metastatic model [312,313]. It is, however, worth to consider that the bursal enclosure of mouse ovary does not apply to human ovary which restricts accurate imitation of metastasis. In vivo monitoring of tumor growth and spreading is repeatedly possible using a bioluminescent marker and detection system. Transferred tumor cells or tumor pieces are stably transfected with luciferase, thereby generating a luminescent signal upon injection of luciferin as a substrate. The signal can be visualized using a bioluminescent imaging technology [314,315,316]. This technique allows in vivo tracking of cells over a long time. Additionally, magnet resonance imaging (MRI) can be used to track cancer cells in mouse models [317,318]. Xenograft models, however, often have the disadvantage of an immune-deficient setting not being able to consider (micro)-environmental tumor interactions. Syngeneic models using genetic engineered murine cell lines for engraftment [319] partially overcome this restriction as applies for humanized mouse models containing human immune cells [307].

Genetically engineered mouse models (GEMMs) for ovarian cancer offer the great chance to precisely predetermine genetic modifications [320,321]. In view of the complex histological and molecular diversity of ovarian cancer it is challenging to establish subtype-mimicking GEMMs that recapitulate geno- and phenotypic features of human cancer. Differentiated models have been developed considering the side of cancer origin (fallopian tube vs. ovarian surface epithelium) as well as tumor genetics targeting critically involved genes (like *Trp53*, *Rb1*, *Pten* or *Dicer1*) and signaling pathways (like PI3K, Myk, PTEN or Wnt/β-catenin) [322,323,324,325,326,327].

Several defined syngeneic models recapitulate specific genetic and epigenetic profiles of various human ovarian cancer subtypes and offer the great opportunity to mimic cancer growth and development as well as drug response [328,329,330]. 

With respect to the exploration of the role of PLKs in cellular biology genetic mouse models using inducible knock-out or knock-in strategies have been valuable as depicted above. A powerful transgenic tool is the inducible RNA interference (RNAi) technology. The *PLK1* gene can be reversibly silenced, as demonstrated in an ovarian cancer cell line, among others [98].

## 7. Conclusions

Taken together, members of the multifunctional and multifaceted PLK family are critically dysregulated in a multitude of malignancies and represent powerful targets in diagnosis and effective therapy. Successful treatment of ovarian cancer remains to be a challenge and especially PLK1 and PLK4 proof to be a very promising target for diagnosis and treatment. Well-suited mouse models are necessary to increase knowledge about tumor biology and to develop powerful therapeutic regimes. Targeting PLKs has the potential for a breakthrough technological advance and clinical success.

## References

[1] Sunkel C.E., Glover D.M. (1988). Polo, a mitotic mutant of Drosophila displaying abnormal spindle poles. J. Cell Sci..

[2] Hamanaka R., Maloid S., Smith M.R., O’Connell C.D., Longo D.L., Ferris D.K. (1994). Cloning and characterization of human and murine homologues of the Drosophila polo serine-threonine kinase. Cell Growth Differ..

[3] Barr F.A., Silljé H.H.W., Nigg E.A. (2004). Polo-like kinases and the orchestration of cell division. Nat. Rev. Mol. Cell Biol..

[4] Lowery D.M., Lim D., Yaffe M.B. (2005). Structure and function of Polo-like kinases. Oncogene.

[5] Van de Weerdt B.C.M., Medema R.H. (2006). Polo-like kinases: A team in control of the division. Cell Cycle.

[6] Zitouni S., Nabais C., Jana S.C., Guerrero A., Bettencourt-Dias M. (2014). Polo-like kinases: Structural variations lead to multiple functions. Nat. Rev. Mol. Cell Biol..

[7] Strebhardt K., Ullrich A. (2006). Targeting polo-like kinase 1 for cancer therapy. Nat. Rev. Cancer.

[8] Colicino E.G., Hehnly H. (2018). Regulating a key mitotic regulator, polo-like kinase 1 (PLK1). Cytoskeleton.

[9] Slevin L.K., Nye J., Pinkerton D.C., Buster D.W., Rogers G.C., Slep K.C. (2012). The structure of the plk4 cryptic polo box reveals two tandem polo boxes required for centriole duplication. Structure.

[10] Maniswami R.R., Prashanth S., Karanth A.V., Koushik S., Govindaraj H., Mullangi R., Rajagopal S., Jegatheesan S.K. (2018). PLK4: A link between centriole biogenesis and cancer. Expert Opin. Ther. Targets.

[11] Garvey D.R., Chhabra G., Ndiaye M.A., Ahmad N. (2021). Role of Polo-Like Kinase 4 (PLK4) in Epithelial Cancers and Recent Progress in its Small Molecule Targeting for Cancer Management. Mol. Cancer Ther..

[12] Lee K.S., Grenfell T.Z., Yarm F.R., Erikson R.L. (1998). Mutation of the polo-box disrupts localization and mitotic functions of the mammalian polo kinase Plk. Proc. Natl. Acad. Sci. USA.

[13] Jang Y.-J., Lin C.-Y., Ma S., Erikson R.L. (2002). Functional studies on the role of the C-terminal domain of mammalian polo-like kinase. Proc. Natl. Acad. Sci. USA.

[14] Elia A.E.H., Rellos P., Haire L.F., Chao J.W., Ivins F.J., Hoepker K., Mohammad D., Cantley L.C., Smerdon S.J., Yaffe M.B. (2003). The molecular basis for phosphodependent substrate targeting and regulation of Plks by the Polo-box domain. Cell.

[15] Archambault V., Lépine G., Kachaner D. (2015). Understanding the Polo Kinase machine. Oncogene.

[16] De Cárcer G., Manning G., Malumbres M. (2011). From Plk1 to Plk5: Functional evolution of polo-like kinases. Cell Cycle.

[17] Strebhardt K. (2010). Multifaceted polo-like kinases: Drug targets and antitargets for cancer therapy. Nat. Rev. Drug Discov..

[18] Schmit T.L., Ahmad N. (2007). Regulation of mitosis via mitotic kinases: New opportunities for cancer management. Mol. Cancer Ther..

[19] Winkles J.A., Alberts G.F. (2005). Differential regulation of polo-like kinase 1, 2, 3, and 4 gene expression in mammalian cells and tissues. Oncogene.

[20] Archambault V., Glover D.M. (2009). Polo-like kinases: Conservation and divergence in their functions and regulation. Nat. Rev. Mol. Cell Biol..

[21] Lu L.-Y., Wood J.L., Minter-Dykhouse K., Ye L., Saunders T.L., Yu X., Chen J. (2008). Polo-like kinase 1 is essential for early embryonic development and tumor suppression. Mol. Cell. Biol..

[22] Wachowicz P., Fernández-Miranda G., Marugán C., Escobar B., de Cárcer G. (2016). Genetic depletion of Polo-like kinase 1 leads to embryonic lethality due to mitotic aberrancies. Bioessays.

[23] Kumar S., Sharma A.R., Sharma G., Chakraborty C., Kim J. (2016). PLK-1: Angel or devil for cell cycle progression. Biochim. Biophys. Acta.

[24] Combes G., Alharbi I., Braga L.G., Elowe S. (2017). Playing polo during mitosis: PLK1 takes the lead. Oncogene.

[25] Seki A., Coppinger J.A., Jang C.-Y., Yates J.R., Fang G. (2008). Bora and the kinase Aurora a cooperatively activate the kinase Plk1 and control mitotic entry. Science.

[26] Eckerdt F., Yuan J., Strebhardt K. (2005). Polo-like kinases and oncogenesis. Oncogene.

[27] Spänkuch B., Steinhauser I., Wartlick H., Kurunci-Csacsko E., Strebhardt K.I., Langer K. (2008). Downregulation of Plk1 expression by receptor-mediated uptake of antisense oligonucleotide-loaded nanoparticles. Neoplasia.

[28] Martin B.T., Strebhardt K. (2006). Polo-like kinase 1: Target and regulator of transcriptional control. Cell Cycle.

[29] Tong C., Fan H.-Y., Lian L., Li S.-W., Chen D.-Y., Schatten H., Sun Q.-Y. (2002). Polo-like kinase-1 is a pivotal regulator of microtubule assembly during mouse oocyte meiotic maturation, fertilization, and early embryonic mitosis. Biol. Reprod.

[30] Solc P., Kitajima T.S., Yoshida S., Brzakova A., Kaido M., Baran V., Mayer A., Samalova P., Motlik J., Ellenberg J. (2015). Multiple requirements of PLK1 during mouse oocyte maturation. PLoS ONE.

[31] Cunningham C.E., MacAuley M.J., Vizeacoumar F.S., Abuhussein O., Freywald A., Vizeacoumar F.J. (2020). The CINs of Polo-Like Kinase 1 in Cancer. Cancers.

[32] Lane H.A., Nigg E.A. (1996). Antibody microinjection reveals an essential role for human polo-like kinase 1 (Plk1) in the functional maturation of mitotic centrosomes. J. Cell Biol..

[33] De Luca M., Lavia P., Guarguaglini G. (2006). A functional interplay between Aurora-A, Plk1 and TPX2 at spindle poles: Plk1 controls centrosomal localization of Aurora-A and TPX2 spindle association. Cell Cycle.

[34] Toyoshima-Morimoto F., Taniguchi E., Shinya N., Iwamatsu A., Nishida E. (2001). Polo-like kinase 1 phosphorylates cyclin B1 and targets it to the nucleus during prophase. Nature.

[35] Toyoshima-Morimoto F., Taniguchi E., Nishida E. (2002). Plk1 promotes nuclear translocation of human Cdc25C during prophase. EMBO Rep..

[36] Gheghiani L., Loew D., Lombard B., Mansfeld J., Gavet O. (2017). PLK1 Activation in Late G2 Sets Up Commitment to Mitosis. Cell Rep..

[37] Sumara I., Giménez-Abián J.F., Gerlich D., Hirota T., Kraft C., de La Torre C., Ellenberg J., Peters J.-M. (2004). Roles of polo-like kinase 1 in the assembly of functional mitotic spindles. Curr. Biol..

[38] Van Vugt M.A.T.M., Brás A., Medema R.H. (2004). Polo-like kinase-1 controls recovery from a G2 DNA damage-induced arrest in mammalian cells. Mol. Cell.

[39] Li H., Liu X.S., Yang X., Wang Y., Wang Y., Turner J.R., Liu X. (2010). Phosphorylation of CLIP-170 by Plk1 and CK2 promotes timely formation of kinetochore-microtubule attachments. EMBO J..

[40] Liu D., Davydenko O., Lampson M.A. (2012). Polo-like kinase-1 regulates kinetochore-microtubule dynamics and spindle checkpoint silencing. J. Cell Biol..

[41] Sumara I., Vorlaufer E., Stukenberg P.T., Kelm O., Redemann N., Nigg E.A., Peters J.-M. (2002). The dissociation of cohesin from chromosomes in prophase is regulated by Polo-like kinase. Mol. Cell.

[42] Kang Y.H., Park J.-E., Yu L.-R., Soung N.-K., Yun S.-M., Bang J.K., Seong Y.-S., Yu H., Garfield S., Veenstra T.D. (2006). Self-regulated Plk1 recruitment to kinetochores by the Plk1-PBIP1 interaction is critical for proper chromosome segregation. Mol. Cell.

[43] Brennan I.M., Peters U., Kapoor T.M., Straight A.F. (2007). Polo-like kinase controls vertebrate spindle elongation and cytokinesis. PLoS ONE.

[44] Petronczki M., Glotzer M., Kraut N., Peters J.-M. (2007). Polo-like kinase 1 triggers the initiation of cytokinesis in human cells by promoting recruitment of the RhoGEF Ect2 to the central spindle. Dev. Cell.

[45] Wolfe B.A., Takaki T., Petronczki M., Glotzer M. (2009). Polo-like kinase 1 directs assembly of the HsCyk-4 RhoGAP/Ect2 RhoGEF complex to initiate cleavage furrow formation. PLoS Biol..

[46] Kumar S., Sharma G., Chakraborty C., Sharma A.R., Kim J. (2017). Regulatory functional territory of PLK-1 and their substrates beyond mitosis. Oncotarget.

[47] Raab C.A., Raab M., Becker S., Strebhardt K. (2021). Non-mitotic functions of polo-like kinases in cancer cells. Biochim. Biophys. Acta Rev. Cancer.

[48] Liu X.S., Li H., Song B., Liu X. (2010). Polo-like kinase 1 phosphorylation of G2 and S-phase-expressed 1 protein is essential for p53 inactivation during G2 checkpoint recovery. EMBO Rep..

[49] Song M.S., Salmena L., Pandolfi P.P. (2012). The functions and regulation of the PTEN tumour suppressor. Nat. Rev. Mol. Cell Biol..

[50] Mandal R., Strebhardt K. (2013). Plk1: Unexpected roles in DNA replication. Cell Res..

[51] Matthess Y., Raab M., Knecht R., Becker S., Strebhardt K. (2014). Sequential Cdk1 and Plk1 phosphorylation of caspase-8 triggers apoptotic cell death during mitosis. Mol. Oncol..

[52] Li Z., Zhang X. (2017). Kinases Involved in Both Autophagy and Mitosis. Int. J. Mol. Sci..

[53] Ruf S., Heberle A.M., Langelaar-Makkinje M., Gelino S., Wilkinson D., Gerbeth C., Schwarz J.J., Holzwarth B., Warscheid B., Meisinger C. (2017). PLK1 (polo like kinase 1) inhibits MTOR complex 1 and promotes autophagy. Autophagy.

[54] Tao Y.-F., Li Z.-H., Du W.-W., Xu L.-X., Ren J.-L., Li X.-L., Fang F., Xie Y., Li M., Qian G.-H. (2017). Inhibiting PLK1 induces autophagy of acute myeloid leukemia cells via mammalian target of rapamycin pathway dephosphorylation. Oncol. Rep..

[55] Simmons D.L., Neel B.G., Stevens R., Evett G., Erikson R.L. (1992). Identification of an early-growth-response gene encoding a novel putative protein kinase. Mol. Cell. Biol..

[56] Liby K., Wu H., Ouyang B., Wu S., Chen J., Dai W. (2001). Identification of the human homologue of the early-growth response gene Snk, encoding a serum-inducible kinase. DNA Seq..

[57] Ma S., Charron J., Erikson R.L. (2003). Role of Plk2 (Snk) in mouse development and cell proliferation. Mol. Cell. Biol..

[58] Villegas E., Kabotyanski E.B., Shore A.N., Creighton C.J., Westbrook T.F., Rosen J.M. (2014). Plk2 regulates mitotic spindle orientation and mammary gland development. Development.

[59] Kauselmann G., Weiler M., Wulff P., Jessberger S., Konietzko U., Scafidi J., Staubli U., Bereiter-Hahn J., Strebhardt K., Kuhl D. (1999). The polo-like protein kinases Fnk and Snk associate with a Ca(2+)- and integrin-binding protein and are regulated dynamically with synaptic plasticity. EMBO J..

[60] Pak D.T.S., Sheng M. (2003). Targeted protein degradation and synapse remodeling by an inducible protein kinase. Science.

[61] Oueslati A., Schneider B.L., Aebischer P., Lashuel H.A. (2013). Polo-like kinase 2 regulates selective autophagic α-synuclein clearance and suppresses its toxicity in vivo. Proc. Natl. Acad. Sci. USA.

[62] Walkup W.G., Sweredoski M.J., Graham R.L., Hess S., Kennedy M.B. (2018). Phosphorylation of synaptic GTPase-activating protein (synGAP) by polo-like kinase (Plk2) alters the ratio of its GAP activity toward HRas, Rap1 and Rap2 GTPases. Biochem. Biophys. Res. Commun..

[63] Seeburg D.P., Feliu-Mojer M., Gaiottino J., Pak D.T.S., Sheng M. (2008). Critical role of CDK5 and Polo-like kinase 2 in homeostatic synaptic plasticity during elevated activity. Neuron.

[64] Warnke S., Kemmler S., Hames R.S., Tsai H.-L., Hoffmann-Rohrer U., Fry A.M., Hoffmann I. (2004). Polo-like kinase-2 is required for centriole duplication in mammalian cells. Curr. Biol..

[65] Cizmecioglu O., Warnke S., Arnold M., Duensing S., Hoffmann I. (2008). Plk2 regulated centriole duplication is dependent on its localization to the centrioles and a functional polo-box domain. Cell Cycle.

[66] Matthew E.M., Yen T.J., Dicker D.T., Dorsey J.F., Yang W., Navaraj A., El-Deiry W.S. (2007). Replication stress, defective S-phase checkpoint and increased death in Plk2-deficient human cancer cells. Cell Cycle.

[67] Burns T.F., Fei P., Scata K.A., Dicker D.T., El-Deiry W.S. (2003). Silencing of the novel p53 target gene Snk/Plk2 leads to mitotic catastrophe in paclitaxel (taxol)-exposed cells. Mol. Cell. Biol..

[68] Yang Y., Bai J., Shen R., Brown S.A.N., Komissarova E., Huang Y., Jiang N., Alberts G.F., Costa M., Lu L. (2008). Polo-like kinase 3 functions as a tumor suppressor and is a negative regulator of hypoxia-inducible factor-1 alpha under hypoxic conditions. Cancer Res..

[69] Myer D.L., Robbins S.B., Yin M., Boivin G.P., Liu Y., Greis K.D., Bahassi E.M., Stambrook P.J. (2011). Absence of polo-like kinase 3 in mice stabilizes Cdc25A after DNA damage but is not sufficient to produce tumors. Mutat. Res..

[70] Wang Q., Xie S., Chen J., Fukasawa K., Naik U., Traganos F., Darzynkiewicz Z., Jhanwar-Uniyal M., Dai W. (2002). Cell cycle arrest and apoptosis induced by human Polo-like kinase 3 is mediated through perturbation of microtubule integrity. Mol. Cell. Biol..

[71] Holtrich U., Wolf G., Yuan J., Bereiter-Hahn J., Karn T., Weiler M., Kauselmann G., Rehli M., Andreesen R., Kaufmann M. (2000). Adhesion induced expression of the serine/threonine kinase Fnk in human macrophages. Oncogene.

[72] Aquino Perez C., Palek M., Stolarova L., von Morgen P., Macurek L. (2020). Phosphorylation of PLK3 Is Controlled by Protein Phosphatase 6. Cells.

[73] Helmke C., Becker S., Strebhardt K. (2016). The role of Plk3 in oncogenesis. Oncogene.

[74] Rödel F., Martin D., Helmke C., Balermpas P., Fokas E., Wieland U., Rave-Fränk M., Kitz J., Matthess Y., Raab M. (2016). Polo-like kinase 3 and phosphoT273 caspase-8 are associated with improved local tumor control and survival in patients with anal carcinoma treated with concomitant chemoradiotherapy. Oncotarget.

[75] Zimmerman W.C., Erikson R.L. (2007). Polo-like kinase 3 is required for entry into S phase. Proc. Natl. Acad. Sci. USA.

[76] Bahassi E.M., Hennigan R.F., Myer D.L., Stambrook P.J. (2004). Cdc25C phosphorylation on serine 191 by Plk3 promotes its nuclear translocation. Oncogene.

[77] Xie S., Wu H., Wang Q., Cogswell J.P., Husain I., Conn C., Stambrook P., Jhanwar-Uniyal M., Dai W. (2001). Plk3 functionally links DNA damage to cell cycle arrest and apoptosis at least in part via the p53 pathway. J. Biol. Chem..

[78] Xie S., Wu H., Wang Q., Kunicki J., Thomas R.O., Hollingsworth R.E., Cogswell J., Dai W. (2002). Genotoxic stress-induced activation of Plk3 is partly mediated by Chk2. Cell Cycle.

[79] Bahassi E.M., Conn C.W., Myer D.L., Hennigan R.F., McGowan C.H., Sanchez Y., Stambrook P.J. (2002). Mammalian Polo-like kinase 3 (Plk3) is a multifunctional protein involved in stress response pathways. Oncogene.

[80] Bahassi E.M., Myer D.L., McKenney R.J., Hennigan R.F., Stambrook P.J. (2006). Priming phosphorylation of Chk2 by polo-like kinase 3 (Plk3) mediates its full activation by ATM and a downstream checkpoint in response to DNA damage. Mutat. Res..

[81] Xu D., Wang Q., Jiang Y., Zhang Y., Vega-Saenzdemiera E., Osman I., Dai W. (2012). Roles of Polo-like kinase 3 in suppressing tumor angiogenesis. Exp. Hematol. Oncol..

[82] Karn T., Holtrich U., Wolf G., Hock B., Strebhardt K., Rubsamenwaigmann H. (1997). Human SAK related to the PLK/polo family of cell cycle kinases shows high mRNA expression in testis. Oncol. Rep..

[83] Hudson J.W., Kozarova A., Cheung P., Macmillan J.C., Swallow C.J., Cross J.C., Dennis J.W. (2001). Late mitotic failure in mice lacking Sak, a polo-like kinase. Curr. Biol..

[84] Habedanck R., Stierhof Y.-D., Wilkinson C.J., Nigg E.A. (2005). The Polo kinase Plk4 functions in centriole duplication. Nat. Cell Biol..

[85] Kleylein-Sohn J., Westendorf J., Le Clech M., Habedanck R., Stierhof Y.-D., Nigg E.A. (2007). Plk4-induced centriole biogenesis in human cells. Dev. Cell.

[86] Sillibourne J.E., Bornens M. (2010). Polo-like kinase 4: The odd one out of the family. Cell Div..

[87] Park J.-E., Zhang L., Bang J.K., Andresson T., DiMaio F., Lee K.S. (2019). Phase separation of Polo-like kinase 4 by autoactivation and clustering drives centriole biogenesis. Nat. Commun..

[88] Breslow D.K., Holland A.J. (2019). Mechanism and Regulation of Centriole and Cilium Biogenesis. Annu. Rev. Biochem..

[89] Zhang X., Wei C., Liang H., Han L. (2021). Polo-Like Kinase 4′s Critical Role in Cancer Development and Strategies for Plk4-Targeted Therapy. Front. Oncol..

[90] Rosario C.O., Ko M.A., Haffani Y.Z., Gladdy R.A., Paderova J., Pollett A., Squire J.A., Dennis J.W., Swallow C.J. (2010). Plk4 is required for cytokinesis and maintenance of chromosomal stability. Proc. Natl. Acad. Sci. USA.

[91] Press M.F., Xie B., Davenport S., Zhou Y., Guzman R., Nolan G.P., O’Brien N., Palazzolo M., Mak T.W., Brugge J.S. (2019). Role for polo-like kinase 4 in mediation of cytokinesis. Proc. Natl. Acad. Sci. USA.

[92] Bonni S., Ganuelas M.L., Petrinac S., Hudson J.W. (2008). Human Plk4 phosphorylates Cdc25C. Cell Cycle.

[93] Montenegro Gouveia S., Zitouni S., Kong D., Duarte P., Ferreira Gomes B., Sousa A.L., Tranfield E.M., Hyman A., Loncarek J., Bettencourt-Dias M. (2018). PLK4 is a microtubule-associated protein that self-assembles promoting de novo MTOC formation. J. Cell Sci..

[94] Byrne D.P., Clarke C.J., Brownridge P.J., Kalyuzhnyy A., Perkins S., Campbell A., Mason D., Jones A.R., Eyers P.A., Eyers C.E. (2020). Use of the Polo-like kinase 4 (PLK4) inhibitor centrinone to investigate intracellular signalling networks using SILAC-based phosphoproteomics. Biochem. J..

[95] Andrysik Z., Bernstein W.Z., Deng L., Myer D.L., Li Y.-Q., Tischfield J.A., Stambrook P.J., Bahassi E.M. (2010). The novel mouse Polo-like kinase 5 responds to DNA damage and localizes in the nucleolus. Nucleic Acids Res..

[96] De Cárcer G., Escobar B., Higuero A.M., García L., Ansón A., Pérez G., Mollejo M., Manning G., Meléndez B., Abad-Rodríguez J. (2011). Plk5, a polo box domain-only protein with specific roles in neuron differentiation and glioblastoma suppression. Mol. Cell. Biol..

[97] Holtrich U., Wolf G., Bräuninger A., Karn T., Böhme B., Rübsamen-Waigmann H., Strebhardt K. (1994). Induction and down-regulation of PLK, a human serine/threonine kinase expressed in proliferating cells and tumors. Proc. Natl. Acad. Sci. USA.

[98] Raab M., Kappel S., Krämer A., Sanhaji M., Matthess Y., Kurunci-Csacsko E., Calzada-Wack J., Rathkolb B., Rozman J., Adler T. (2011). Toxicity modelling of Plk1-targeted therapies in genetically engineered mice and cultured primary mammalian cells. Nat. Commun..

[99] Raab M., Sanhaji M., Matthess Y., Hörlin A., Lorenz I., Dötsch C., Habbe N., Waidmann O., Kurunci-Csacsko E., Firestein R. (2018). PLK1 has tumor-suppressive potential in APC-truncated colon cancer cells. Nat. Commun..

[100] De Cárcer G., Venkateswaran S.V., Salgueiro L., El Bakkali A., Somogyi K., Rowald K., Montañés P., Sanclemente M., Escobar B., de Martino A. (2018). Plk1 overexpression induces chromosomal instability and suppresses tumor development. Nat. Commun..

[101] Smith M.R., Wilson M.L., Hamanaka R., Chase D., Kung H., Longo D.L., Ferris D.K. (1997). Malignant transformation of mammalian cells initiated by constitutive expression of the polo-like kinase. Biochem. Biophys. Res. Commun..

[102] Liu Z., Sun Q., Wang X. (2017). PLK1, A Potential Target for Cancer Therapy. Transl. Oncol..

[103] Wolf G., Elez R., Doermer A., Holtrich U., Ackermann H., Stutte H.J., Altmannsberger H.M., Rübsamen-Waigmann H., Strebhardt K. (1997). Prognostic significance of polo-like kinase (PLK) expression in non-small cell lung cancer. Oncogene.

[104] Weichert W., Kristiansen G., Winzer K.-J., Schmidt M., Gekeler V., Noske A., Müller B.-M., Niesporek S., Dietel M., Denkert C. (2005). Polo-like kinase isoforms in breast cancer: Expression patterns and prognostic implications. Virchows Arch..

[105] Li H., Wang H., Sun Z., Guo Q., Shi H., Jia Y. (2017). The clinical and prognostic value of polo-like kinase 1 in lung squamous cell carcinoma patients: Immunohistochemical analysis. Biosci. Rep..

[106] Tokumitsu Y., Mori M., Tanaka S., Akazawa K., Nakano S., Niho Y. (1999). Prognostic significance of polo-like kinase expression in esophageal carcinoma. Int. J. Oncol..

[107] Lan B., Liu B.-Y., Chen X.-H., Qu Y., Zhang X.-Q., Cai Q., Zhu Z.-G. (2007). Polo like kinase 1 expression and prognostic value in gastric carcinomas. Zhonghua Wei Chang. Wai Ke Za Zhi.

[108] Feng Y.-B., Lin D.-C., Shi Z.-Z., Wang X.-C., Shen X.-M., Zhang Y., Du X.-L., Luo M.-L., Xu X., Han Y.-L. (2009). Overexpression of PLK1 is associated with poor survival by inhibiting apoptosis via enhancement of survivin level in esophageal squamous cell carcinoma. Int. J. Cancer.

[109] Knecht R., Elez R., Oechler M., Solbach C., von Ilberg C., Strebhardt K. (1999). Prognostic significance of polo-like kinase (PLK) expression in squamous cell carcinomas of the head and neck. Cancer Res..

[110] Knecht R., Oberhauser C., Strebhardt K. (2000). PLK (polo-like kinase), a new prognostic marker for oropharyngeal carcinomas. Int. J. Cancer.

[111] King S.I., Purdie C.A., Bray S.E., Quinlan P.R., Jordan L.B., Thompson A.M., Meek D.W. (2012). Immunohistochemical detection of Polo-like kinase-1 (PLK1) in primary breast cancer is associated with TP53 mutation and poor clinical outcom. Breast Cancer Res..

[112] Ren D., Hua Y., Yu B., Ye X., He Z., Li C., Wang J., Mo Y., Wei X., Chen Y. (2020). Predictive biomarkers and mechanisms underlying resistance to PD1/PD-L1 blockade cancer immunotherapy. Mol. Cancer.

[113] Strebhardt K., Kneisel L., Linhart C., Bernd A., Kaufmann R. (2000). Prognostic value of pololike kinase expression in melanomas. JAMA.

[114] Amani V., Prince E.W., Alimova I., Balakrishnan I., Birks D., Donson A.M., Harris P., Levy J.M.M., Handler M., Foreman N.K. (2016). Polo-like Kinase 1 as a potential therapeutic target in Diffuse Intrinsic Pontine Glioma. BMC Cancer.

[115] Schmit T.L., Zhong W., Setaluri V., Spiegelman V.S., Ahmad N. (2009). Targeted depletion of Polo-like kinase (Plk) 1 through lentiviral shRNA or a small-molecule inhibitor causes mitotic catastrophe and induction of apoptosis in human melanoma cells. J. Investig. Dermatol..

[116] Jalili A., Moser A., Pashenkov M., Wagner C., Pathria G., Borgdorff V., Gschaider M., Stingl G., Ramaswamy S., Wagner S.N. (2011). Polo-like kinase 1 is a potential therapeutic target in human melanoma. J. Investig. Dermatol..

[117] Schmit T.L., Zhong W., Nihal M., Ahmad N. (2009). Polo-like kinase 1 (Plk1) in non-melanoma skin cancers. Cell Cycle.

[118] Takahashi T., Sano B., Nagata T., Kato H., Sugiyama Y., Kunieda K., Kimura M., Okano Y., Saji S. (2003). Polo-like kinase 1 (PLK1) is overexpressed in primary colorectal cancers. Cancer Sci..

[119] Han D.-P., Zhu Q.-L., Cui J.-T., Wang P.-X., Qu S., Cao Q.-F., Zong Y.-P., Feng B., Zheng M.-H., Lu A.-G. (2012). Polo-like kinase 1 is overexpressed in colorectal cancer and participates in the migration and invasion of colorectal cancer cells. Med. Sci. Monit..

[120] Tut T.G., Lim S.H.S., Dissanayake I.U., Descallar J., Chua W., Ng W., de Souza P., Shin J.-S., Lee C.S. (2015). Upregulated Polo-Like Kinase 1 Expression Correlates with Inferior Survival Outcomes in Rectal Cancer. PLoS ONE.

[121] Rödel F., Keppner S., Capalbo G., Bashary R., Kaufmann M., Rödel C., Strebhardt K., Spänkuch B. (2010). Polo-like kinase 1 as predictive marker and therapeutic target for radiotherapy in rectal cancer. Am. J. Pathol..

[122] Triscott J., Lee C., Foster C., Manoranjan B., Pambid M.R., Berns R., Fotovati A., Venugopal C., O’Halloran K., Narendran A. (2013). Personalizing the treatment of pediatric medulloblastoma: Polo-like kinase 1 as a molecular target in high-risk children. Cancer Res..

[123] Linton A., Cheng Y.Y., Griggs K., Schedlich L., Kirschner M.B., Gattani S., Srikaran S., Chuan-Hao Kao S., McCaughan B.C., Klebe S. (2014). An RNAi-based screen reveals PLK1, CDK1 and NDC80 as potential therapeutic targets in malignant pleural mesothelioma. Br. J. Cancer.

[124] Dietzmann K., Kirches E., Mawrin C. (2002). Effects of phospholipase Cgamma on Polo-like kinase 1 expression in human glioma cells. J. Cancer Res. Clin. Oncol..

[125] Li Z., Yang C., Li X., Du X., Tao Y., Ren J., Fang F., Xie Y., Li M., Qian G. (2020). The dual role of BI 2536, a small-molecule inhibitor that targets PLK1, in induction of apoptosis and attenuation of autophagy in neuroblastoma cells. J. Cancer.

[126] Ackermann S., Goeser F., Schulte J.H., Schramm A., Ehemann V., Hero B., Eggert A., Berthold F., Fischer M. (2011). Polo-like kinase 1 is a therapeutic target in high-risk neuroblastoma. Clin. Cancer Res..

[127] Takai N., Hamanaka R., Yoshimatsu J., Miyakawa I. (2005). Polo-like kinases (Plks) and cancer. Oncogene.

[128] Ito Y., Yoshida H., Matsuzuka F., Matsuura N., Nakamura Y., Nakamine H., Kakudo K., Kuma K., Miyauchi A. (2004). Polo-like kinase 1 (PLK1) expression is associated with cell proliferative activity and cdc2 expression in malignant lymphoma of the thyroid. Anticancer Res..

[129] Gray P.J., Bearss D.J., Han H., Nagle R., Tsao M.-S., Dean N., von Hoff D.D. (2004). Identification of human polo-like kinase 1 as a potential therapeutic target in pancreatic cancer. Mol. Cancer Ther..

[130] Weichert W., Schmidt M., Gekeler V., Denkert C., Stephan C., Jung K., Loening S., Dietel M., Kristiansen G. (2004). Polo-like kinase 1 is overexpressed in prostate cancer and linked to higher tumor grades. Prostate.

[131] Reagan-Shaw S., Ahmad N. (2005). Polo-like kinase (Plk) 1 as a target for prostate cancer management. IUBMB Life.

[132] Yamada S.-i., Ohira M., Horie H., Ando K., Takayasu H., Suzuki Y., Sugano S., Hirata T., Goto T., Matsunaga T. (2004). Expression profiling and differential screening between hepatoblastomas and the corresponding normal livers: Identification of high expression of the PLK1 oncogene as a poor-prognostic indicator of hepatoblastomas. Oncogene.

[133] Wang X.Q., Zhu Y.Q., Lui K.S., Cai Q., Lu P., Poon R.T. (2008). Aberrant Polo-like kinase 1-Cdc25A pathway in metastatic hepatocellular carcinoma. Clin. Cancer Res..

[134] Lin P., Wen D.-Y., Dang Y.-W., He Y., Yang H., Chen G. (2018). Comprehensive and Integrative Analysis Reveals the Diagnostic, Clinicopathological and Prognostic Significance of Polo-Like Kinase 1 in Hepatocellular Carcinoma. Cell. Physiol. Biochem..

[135] Yousef E.H., El-Mesery M.E., Habeeb M.R., Eissa L.A. (2020). Polo-like kinase 1 as a promising diagnostic biomarker and potential therapeutic target for hepatocellular carcinoma. Tumor Biol..

[136] Mito K., Kashima K., Kikuchi H., Daa T., Nakayama I., Yokoyama S. (2005). Expression of Polo-Like Kinase (PLK1) in non-Hodgkin’s lymphomas. Leuk. Lymphoma.

[137] Stutz N., Nihal M., Wood G.S. (2011). Polo-like kinase 1 (Plk1) in cutaneous T-cell lymphoma. Br. J. Dermatol..

[138] Zhang G., Zhang Z., Liu Z. (2013). Polo-like kinase 1 is overexpressed in renal cancer and participates in the proliferation and invasion of renal cancer cells. Tumor Biol..

[139] Zhang Z., Zhang G., Kong C. (2013). High expression of polo-like kinase 1 is associated with the metastasis and recurrence in urothelial carcinoma of bladder. Urol. Oncol..

[140] Lake R.J., Jelinek W.R. (1993). Cell cycle- and terminal differentiation-associated regulation of the mouse mRNA encoding a conserved mitotic protein kinase. Mol. Cell. Biol..

[141] Golsteyn R.M., Schultz S.J., Bartek J., Ziemiecki A., Ried T., Nigg E.A. (1994). Cell cycle analysis and chromosomal localization of human Plk1, a putative homologue of the mitotic kinases Drosophila polo and Saccharomyces cerevisiae Cdc5. J. Cell Sci..

[142] Matsubara N., Yanagisawa M., Nishimune Y., Obinata M., Matsui Y. (1995). Murine polo like kinase 1 gene is expressed in meiotic testicular germ cells and oocytes. Mol. Reprod. Dev..

[143] Takai N., Yoshimatsu J., Nishida Y., Narahara H., Miyakawa I., Hamanaka R. (1999). Expression of polo-like kinase (PLK) in the mouse placenta and ovary. Reprod. Fertil. Dev..

[144] Rödel F., Zhou S., Győrffy B., Raab M., Sanhaji M., Mandal R., Martin D., Becker S., Strebhardt K. (2020). The Prognostic Relevance of the Proliferation Markers Ki-67 and Plk1 in Early-Stage Ovarian Cancer Patients With Serous, Low-Grade Carcinoma Based on mRNA and Protein Expression. Front. Oncol..

[145] Kaczorowski M., Borowiec T., Donizy P., Pagacz K., Fendler W., Lipinski A., Halon A., Matkowski R. (2017). Polo-like kinase-1 immunoreactivity is associated with metastases in cutaneous melanoma. J. Cutan. Pathol..

[146] Cholewa B.D., Liu X., Ahmad N. (2013). The role of polo-like kinase 1 in carcinogenesis: Cause or consequence?. Cancer Res..

[147] Fu Z., Wen D. (2017). The Emerging Role of Polo-Like Kinase 1 in Epithelial-Mesenchymal Transition and Tumor Metastasis. Cancers.

[148] Gutteridge R.E.A., Ndiaye M.A., Liu X., Ahmad N. (2016). Plk1 Inhibitors in Cancer Therapy: From Laboratory to Clinics. Mol. Cancer Ther..

[149] De Cárcer G. (2019). The Mitotic Cancer Target Polo-Like Kinase 1: Oncogene or Tumor Suppressor?. Genes.

[150] Louwen F., Yuan J. (2013). Battle of the eternal rivals: Restoring functional p53 and inhibiting Polo-like kinase 1 as cancer therapy. Oncotarget.

[151] Nigro J.M., Baker S.J., Preisinger A.C., Jessup J.M., Hostetter R., Cleary K., Bigner S.H., Davidson N., Baylin S., Devilee P. (1989). Mutations in the p53 gene occur in diverse human tumour types. Nature.

[152] Hainaut P., Hollstein M. (2000). p53 and human cancer: The first ten thousand mutations. Adv. Cancer Res..

[153] Ren Y., Bi C., Zhao X., Lwin T., Wang C., Yuan J., Silva A.S., Shah B.D., Fang B., Li T. (2018). PLK1 stabilizes a MYC-dependent kinase network in aggressive B cell lymphomas. J. Clin. Investig..

[154] Zhang R., Shi H., Ren F., Liu H., Zhang M., Deng Y., Li X. (2015). Misregulation of polo-like protein kinase 1, P53 and P21WAF1 in epithelial ovarian cancer suggests poor prognosis. Oncol. Rep..

[155] Li Z., Li J., Bi P., Lu Y., Burcham G., Elzey B.D., Ratliff T., Konieczny S.F., Ahmad N., Kuang S. (2014). Plk1 phosphorylation of PTEN causes a tumor-promoting metabolic state. Mol. Cell. Biol..

[156] Lee J., Lee J., Sim W., Kim J.-H. (2020). Differential Dependency of Human Pancreatic Cancer Cells on Targeting PTEN via PLK 1 Expression. Cancers.

[157] Gutteridge R.E.A., Singh C.K., Ndiaye M.A., Ahmad N. (2017). Targeted knockdown of polo-like kinase 1 alters metabolic regulation in melanoma. Cancer Lett..

[158] Ma X., Wang L., Huang D., Li Y., Yang D., Li T., Li F., Sun L., Wei H., He K. (2017). Polo-like kinase 1 coordinates biosynthesis during cell cycle progression by directly activating pentose phosphate pathway. Nat. Commun..

[159] Morandi A., Taddei M.L., Chiarugi P., Giannoni E. (2017). Targeting the Metabolic Reprogramming That Controls Epithelial-to-Mesenchymal Transition in Aggressive Tumors. Front. Oncol..

[160] Kalluri R., Weinberg R.A. (2009). The basics of epithelial-mesenchymal transition. J. Clin. Investig..

[161] Yamaguchi H., Condeelis J. (2007). Regulation of the actin cytoskeleton in cancer cell migration and invasion. Biochim. Biophys. Acta.

[162] Friedl P., Alexander S. (2011). Cancer invasion and the microenvironment: Plasticity and reciprocity. Cell.

[163] Fu Z., Malureanu L., Huang J., Wang W., Li H., van Deursen J.M., Tindall D.J., Chen J. (2008). Plk1-dependent phosphorylation of FoxM1 regulates a transcriptional programme required for mitotic progression. Nat. Cell Biol..

[164] Wu J., Ivanov A.I., Fisher P.B., Fu Z. (2016). Polo-like kinase 1 induces epithelial-to-mesenchymal transition and promotes epithelial cell motility by activating CRAF/ERK signaling. Elife.

[165] Cai X.P., Chen L.D., Song H.B., Zhang C.X., Yuan Z.W., Xiang Z.X. (2016). PLK1 promotes epithelial-mesenchymal transition and metastasis of gastric carcinoma cells. Am. J. Transl. Res..

[166] De Cárcer G., Wachowicz P., Martínez-Martínez S., Oller J., Méndez-Barbero N., Escobar B., González-Loyola A., Takaki T., El Bakkali A., Cámara J.A. (2017). Plk1 regulates contraction of postmitotic smooth muscle cells and is required for vascular homeostasis. Nat. Med..

[167] Xie Y., Liu Y., Li Q., Chen J. (2018). Polo-like kinase 2 promotes chemoresistance and predicts limited survival benefit from adjuvant chemotherapy in colorectal cancer. Int. J. Oncol..

[168] Alafate W., Xu D., Wu W., Xiang J., Ma X., Xie W., Bai X., Wang M., Wang J. (2020). Loss of PLK2 induces acquired resistance to temozolomide in GBM via activation of notch signaling. J. Exp. Clin. Cancer Res..

[169] Pellegrino R., Calvisi D.F., Ladu S., Ehemann V., Staniscia T., Evert M., Dombrowski F., Schirmacher P., Longerich T. (2010). Oncogenic and tumor suppressive roles of polo-like kinases in human hepatocellular carcinoma. Hepatology.

[170] Benetatos L., Dasoula A., Hatzimichael E., Syed N., Voukelatou M., Dranitsaris G., Bourantas K.L., Crook T. (2011). Polo-like kinase 2 (SNK/PLK2) is a novel epigenetically regulated gene in acute myeloid leukemia and myelodysplastic syndromes: Genetic and epigenetic interactions. Ann. Hematol..

[171] Syed N., Smith P., Sullivan A., Spender L.C., Dyer M., Karran L., O’Nions J., Allday M., Hoffmann I., Crawford D. (2006). Transcriptional silencing of Polo-like kinase 2 (SNK/PLK2) is a frequent event in B-cell malignancies. Blood.

[172] Liu F., Zhang S., Zhao Z., Mao X., Huang J., Wu Z., Zheng L., Wang Q. (2016). MicroRNA-27b up-regulated by human papillomavirus 16 E7 promotes proliferation and suppresses apoptosis by targeting polo-like kinase2 in cervical cancer. Oncotarget.

[173] Liu L.Y., Wang W., Zhao L.Y., Guo B., Yang J., Zhao X.G., Song T.S., Huang C., Xu J.R. (2015). Silencing of polo-like kinase 2 increases cell proliferation and decreases apoptosis in SGC-7901 gastric cancer cells. Mol. Med. Rep..

[174] Gee H.E., Buffa F.M., Harris A.L., Toohey J.M., Carroll S.L., Cooper C.L., Beith J., McNeil C., Carmalt H., Mak C. (2015). MicroRNA-Related DNA Repair/Cell-Cycle Genes Independently Associated With Relapse After Radiation Therapy for Early Breast Cancer. Int. J. Radiat. Oncol. Biol. Phys..

[175] Matthew E.M., Yang Z., Peri S., Andrake M., Dunbrack R., Ross E., El-Deiry W.S. (2018). Plk2 Loss Commonly Occurs in Colorectal Carcinomas but not Adenomas: Relationship to mTOR Signaling. Neoplasia.

[176] Vogelstein B., Kinzler K.W. (1992). p53 function and dysfunction. Cell.

[177] Ou B., Zhao J., Guan S., Wangpu X., Zhu C., Zong Y., Ma J., Sun J., Zheng M., Feng H. (2016). Plk2 promotes tumor growth and inhibits apoptosis by targeting Fbxw7/Cyclin E in colorectal cancer. Cancer Lett..

[178] Han T., Lin J., Wang Y., Fan Q., Sun H., Tao Y., Sun C. (2018). Forkhead box D1 promotes proliferation and suppresses apoptosis via regulating polo-like kinase 2 in colorectal cancer. Biomed. Pharmacother..

[179] Shen T., Li Y., Yang L., Xu X., Liang F., Liang S., Ba G., Xue F., Fu Q. (2012). Upregulation of Polo-like kinase 2 gene expression by GATA-1 acetylation in human osteosarcoma MG-63 cells. Int. J. Biochem. Cell Biol..

[180] Fingas C.D., Mertens J.C., Razumilava N., Sydor S., Bronk S.F., Christensen J.D., Rizvi S.H., Canbay A., Treckmann J.W., Paul A. (2013). Polo-like kinase 2 is a mediator of hedgehog survival signaling in cholangiocarcinoma. Hepatology.

[181] Hu Z., Xu Z., Liao X., Yang X., Dong C., Luk K., Jin A., Lu H. (2015). Polo-like kinase 2 acting as a promoter in human tumor cells with an abundance of TAp73. OncoTargets Ther..

[182] Li W., Zhang X., Xi X., Li Y., Quan H., Liu S., Wu L., Wu P., Lan W., Shao Y. (2020). PLK2 modulation of enriched TAp73 affects osteogenic differentiation and prognosis in human osteosarcoma. Cancer Med..

[183] Kothari V., Wei I., Shankar S., Kalyana-Sundaram S., Wang L., Ma L.W., Vats P., Grasso C.S., Robinson D.R., Wu Y.-M. (2013). Outlier kinase expression by RNA sequencing as targets for precision therapy. Cancer Discov..

[184] Lee J.S., Lee Y., André E.A., Lee K.J., Nguyen T., Feng Y., Jia N., Harris B.T., Burns M.P., Pak D.T.S. (2019). Inhibition of Polo-like kinase 2 ameliorates pathogenesis in Alzheimer’s disease model mice. PLoS ONE.

[185] Li B., Ouyang B., Pan H., Reissmann P.T., Slamon D.J., Arceci R., Lu L., Dai W. (1996). Prk, a cytokine-inducible human protein serine/threonine kinase whose expression appears to be down-regulated in lung carcinomas. J. Biol. Chem..

[186] Ando K., Ozaki T., Yamamoto H., Furuya K., Hosoda M., Hayashi S., Fukuzawa M., Nakagawara A. (2004). Polo-like kinase 1 (Plk1) inhibits p53 function by physical interaction and phosphorylation. J. Biol. Chem..

[187] Dai W., Li Y., Ouyang B., Pan H., Reissmann P., Li J., Wiest J., Stambrook P., Gluckman J.L., Noffsinger A. (2000). PRK, a cell cycle gene localized to 8p21, is downregulated in head and neck cancer. Genes Chromosomes Cancer.

[188] Dai W., Liu T., Wang Q., Rao C.V., Reddy B.S. (2002). Down-regulation of PLK3 gene expression by types and amount of dietary fat in rat colon tumors. Int. J. Oncol..

[189] Lin C., Bai S., Du T., Lai Y., Chen X., Peng S., Ma X., Wu W., Guo Z., Huang H. (2019). Polo-like kinase 3 is associated with poor prognosis and regulates proliferation and metastasis in prostate cancer. Cancer Manag. Res..

[190] Fleischmann M., Martin D., Peña-Llopis S., Oppermann J., von der Grün J., Diefenhardt M., Chatzikonstantinou G., Fokas E., Rödel C., Strebhardt K. (2019). Association of Polo-Like Kinase 3 and PhosphoT273 Caspase 8 Levels With Disease-Related Outcomes Among Cervical Squamous Cell Carcinoma Patients Treated With Chemoradiation and Brachytherapy. Front. Oncol..

[191] Harris A.L. (2002). Hypoxia—A key regulatory factor in tumour growth. Nat. Rev. Cancer.

[192] Xu D., Yao Y., Lu L., Costa M., Dai W. (2010). Plk3 functions as an essential component of the hypoxia regulatory pathway by direct phosphorylation of HIF-1alpha. J. Biol. Chem..

[193] Xu D., Dai W., Li C. (2017). Polo-like kinase 3, hypoxic responses, and tumorigenesis. Cell Cycle.

[194] Jen K.-Y., Cheung V.G. (2005). Identification of novel p53 target genes in ionizing radiation response. Cancer Res..

[195] Li Z., Niu J., Uwagawa T., Peng B., Chiao P.J. (2005). Function of polo-like kinase 3 in NF-kappaB-mediated proapoptotic response. J. Biol. Chem..

[196] Finetti P., Cervera N., Charafe-Jauffret E., Chabannon C., Charpin C., Chaffanet M., Jacquemier J., Viens P., Birnbaum D., Bertucci F. (2008). Sixteen-kinase gene expression identifies luminal breast cancers with poor prognosis. Cancer Res..

[197] Marina M., Saavedra H.I. (2014). Nek2 and Plk4: Prognostic markers, drivers of breast tumorigenesis and drug resistance. Front. Biosci..

[198] Denu R.A., Zasadil L.M., Kanugh C., Laffin J., Weaver B.A., Burkard M.E. (2016). Centrosome amplification induces high grade features and is prognostic of worse outcomes in breast cancer. BMC Cancer.

[199] Li Z., Dai K., Wang C., Song Y., Gu F., Liu F., Fu L. (2016). Expression of Polo-Like Kinase 4(PLK4) in Breast Cancer and Its Response to Taxane-Based Neoadjuvant Chemotherapy. J. Cancer.

[200] Kawakami M., Mustachio L.M., Zheng L., Chen Y., Rodriguez-Canales J., Mino B., Kurie J.M., Roszik J., Villalobos P.A., Thu K.L. (2018). Polo-like kinase 4 inhibition produces polyploidy and apoptotic death of lung cancers. Proc. Natl. Acad. Sci. USA.

[201] Zhou Q., Fan G., Dong Y. (2020). Polo-like kinase 4 correlates with greater tumor size, lymph node metastasis and confers poor survival in non-small cell lung cancer. J. Clin. Lab. Anal..

[202] Korzeniewski N., Hohenfellner M., Duensing S. (2012). CAND1 promotes PLK4-mediated centriole overduplication and is frequently disrupted in prostate cancer. Neoplasia.

[203] Shinmura K., Kurabe N., Goto M., Yamada H., Natsume H., Konno H., Sugimura H. (2014). PLK4 overexpression and its effect on centrosome regulation and chromosome stability in human gastric cancer. Mol. Biol. Rep..

[204] Cao T., Yi S., Yang X., Wu Q. (2020). Clinical Significance of Polo-Like Kinase 4 as a Marker for Advanced Tumor Stage and Dismal Prognosis in Patients With Surgical Gastric Cancer. Technol. Cancer Res. Treat..

[205] Tian X., Zhou D., Chen L., Tian Y., Zhong B., Cao Y., Dong Q., Zhou M., Yan J., Wang Y. (2018). Polo-like kinase 4 mediates epithelial-mesenchymal transition in neuroblastoma via PI3K/Akt signaling pathway. Cell Death Dis..

[206] Wang J., Zuo J., Wang M., Ma X., Gao K., Bai X., Wang N., Xie W., Liu H. (2019). Polo-like kinase 4 promotes tumorigenesis and induces resistance to radiotherapy in glioblastoma. Oncol. Rep..

[207] Goroshchuk O., Kolosenko I., Vidarsdottir L., Azimi A., Palm-Apergi C. (2019). Polo-like kinases and acute leukemia. Oncogene.

[208] Goroshchuk O., Vidarsdottir L., Björklund A.-C., Hamil A.S., Kolosenko I., Dowdy S.F., Palm-Apergi C. (2020). Targeting Plk1 with siRNNs in primary cells from pediatric B-cell acute lymphoblastic leukemia patients. Sci. Rep..

[209] Coelho P.A., Bury L., Shahbazi M.N., Liakath-Ali K., Tate P.H., Wormald S., Hindley C.J., Huch M., Archer J., Skarnes W.C. (2015). Over-expression of Plk4 induces centrosome amplification, loss of primary cilia and associated tissue hyperplasia in the mouse. Open Biol..

[210] Serçin Ö., Larsimont J.-C., Karambelas A.E., Marthiens V., Moers V., Boeckx B., Le Mercier M., Lambrechts D., Basto R., Blanpain C. (2016). Transient PLK4 overexpression accelerates tumorigenesis in p53-deficient epidermis. Nat. Cell Biol..

[211] Denu R.A., Shabbir M., Nihal M., Singh C.K., Longley B.J., Burkard M.E., Ahmad N. (2018). Centriole Overduplication is the Predominant Mechanism Leading to Centrosome Amplification in Melanoma. Mol. Cancer Res..

[212] Macmillan J.C., Hudson J.W., Bull S., Dennis J.W., Swallow C.J. (2001). Comparative expression of the mitotic regulators SAK and PLK in colorectal cancer. Ann. Surg. Oncol..

[213] Bao J., Yu Y., Chen J., He Y., Chen X., Ren Z., Xue C., Liu L., Hu Q., Li J. (2018). MiR-126 negatively regulates PLK-4 to impact the development of hepatocellular carcinoma via ATR/CHEK1 pathway. Cell Death Dis..

[214] Liao Z., Zhang H., Fan P., Huang Q., Dong K., Qi Y., Song J., Chen L., Liang H., Chen X. (2019). High PLK4 expression promotes tumor progression and induces epithelial-mesenchymal transition by regulating the Wnt/β-catenin signaling pathway in colorectal cancer. Int. J. Oncol..

[215] Ko M.A., Rosario C.O., Hudson J.W., Kulkarni S., Pollett A., Dennis J.W., Swallow C.J. (2005). Plk4 haploinsufficiency causes mitotic infidelity and carcinogenesis. Nat. Genet..

[216] Schukken K.M., Foijer F. (2018). CIN and Aneuploidy: Different Concepts, Different Consequences. Bioessays.

[217] Holland A.J., Cleveland D.W. (2014). Polo-like kinase 4 inhibition: A strategy for cancer therapy?. Cancer Cell.

[218] Kops G.J.P.L., Weaver B.A.A., Cleveland D.W. (2005). On the road to cancer: Aneuploidy and the mitotic checkpoint. Nat. Rev. Cancer.

[219] Susini T., Olivieri S., Molino C., Amunni G., Rapi S., Taddei G., Scarselli G. (2011). DNA ploidy is stronger than lymph node metastasis as prognostic factor in cervical carcinoma: 10-year results of a prospective study. Int. J. Gynecol. Cancer.

[220] Braun M., Stomper J., Kirsten R., Shaikhibrahim Z., Vogel W., Böhm D., Wernert N., Kristiansen G., Perner S. (2013). Landscape of chromosome number changes in prostate cancer progression. World J. Urol..

[221] Xu J., Huang L., Li J. (2016). DNA aneuploidy and breast cancer: A meta-analysis of 141,163 cases. Oncotarget.

[222] Levine M.S., Bakker B., Boeckx B., Moyett J., Lu J., Vitre B., Spierings D.C., Lansdorp P.M., Cleveland D.W., Lambrechts D. (2017). Centrosome Amplification Is Sufficient to Promote Spontaneous Tumorigenesis in Mammals. Dev. Cell.

[223] Godinho S.A., Picone R., Burute M., Dagher R., Su Y., Leung C.T., Polyak K., Brugge J.S., Théry M., Pellman D. (2014). Oncogene-like induction of cellular invasion from centrosome amplification. Nature.

[224] Rosario C.O., Kazazian K., Zih F.S.W., Brashavitskaya O., Haffani Y., Xu R.S.Z., George A., Dennis J.W., Swallow C.J. (2015). A novel role for Plk4 in regulating cell spreading and motility. Oncogene.

[225] Kazazian K., Go C., Wu H., Brashavitskaya O., Xu R., Dennis J.W., Gingras A.-C., Swallow C.J. (2017). Plk4 Promotes Cancer Invasion and Metastasis through Arp2/3 Complex Regulation of the Actin Cytoskeleton. Cancer Res..

[226] Liu Y., Kim J., Philip R., Sridhar V., Chandrashekhar M., Moffat J., van Breugel M., Pelletier L. (2020). Direct interaction between CEP85 and STIL mediates PLK4-driven directed cell migration. J. Cell Sci..

[227] Luo Y., Barrios-Rodiles M., Gupta G.D., Zhang Y.Y., Ogunjimi A.A., Bashkurov M., Tkach J.M., Underhill A.Q., Zhang L., Bourmoum M. (2019). Atypical function of a centrosomal module in WNT signalling drives contextual cancer cell motility. Nat. Commun..

[228] Li Z., Hao P., Wu Q., Li F., Zhao J., Wu K., Qu C., Chen Y., Li M., Chen X. (2016). Genetic mutations associated with metastatic clear cell renal cell carcinoma. Oncotarget.

[229] Zhao Y., Wang X. (2019). PLK4: A promising target for cancer therapy. J. Cancer Res. Clin. Oncol..

[230] Spänkuch-Schmitt B., Bereiter-Hahn J., Kaufmann M., Strebhardt K. (2002). Effect of RNA silencing of polo-like kinase-1 (PLK1) on apoptosis and spindle formation in human cancer cells. J. Natl. Cancer Inst..

[231] Spänkuch-Schmitt B., Wolf G., Solbach C., Loibl S., Knecht R., Stegmüller M., von Minckwitz G., Kaufmann M., Strebhardt K. (2002). Downregulation of human polo-like kinase activity by antisense oligonucleotides induces growth inhibition in cancer cells. Oncogene.

[232] Spänkuch B., Matthess Y., Knecht R., Zimmer B., Kaufmann M., Strebhardt K. (2004). Cancer inhibition in nude mice after systemic application of U6 promoter-driven short hairpin RNAs against PLK1. J. Natl. Cancer Inst..

[233] Cholewa B.D., Ndiaye M.A., Huang W., Liu X., Ahmad N. (2017). Small molecule inhibition of polo-like kinase 1 by volasertib (BI 6727) causes significant melanoma growth delay and regression in vivo. Cancer Lett..

[234] Murugan R.N., Ahn M., Lee W.C., Kim H.-Y., Song J.H., Cheong C., Hwang E., Seo J.-H., Shin S.Y., Choi S.H. (2013). Exploring the binding nature of pyrrolidine pocket-dependent interactions in the polo-box domain of polo-like kinase 1. PLoS ONE.

[235] Schmit T.L., Ledesma M.C., Ahmad N. (2010). Modulating polo-like kinase 1 as a means for cancer chemoprevention. Pharm. Res..

[236] Li J., Karki A., Hodges K.B., Ahmad N., Zoubeidi A., Strebhardt K., Ratliff T.L., Konieczny S.F., Liu X. (2015). Cotargeting Polo-Like Kinase 1 and the Wnt/β-Catenin Signaling Pathway in Castration-Resistant Prostate Cancer. Mol. Cell. Biol..

[237] Liu X. (2015). Targeting Polo-Like Kinases: A Promising Therapeutic Approach for Cancer Treatment. Transl. Oncol..

[238] Palmisiano N.D., Kasner M.T. (2015). Polo-like kinase and its inhibitors: Ready for the match to start?. Am. J. Hematol..

[239] Kumar S., Kim J. (2015). PLK-1 Targeted Inhibitors and Their Potential against Tumorigenesis. BioMed Res. Int..

[240] Lee K.S., Burke T.R., Park J.-E., Bang J.K., Lee E. (2015). Recent Advances and New Strategies in Targeting Plk1 for Anticancer Therapy. Trends Pharmacol. Sci..

[241] Hu C.-K., Ozlü N., Coughlin M., Steen J.J., Mitchison T.J. (2012). Plk1 negatively regulates PRC1 to prevent premature midzone formation before cytokinesis. Mol. Biol. Cell.

[242] Raab M., Krämer A., Hehlgans S., Sanhaji M., Kurunci-Csacsko E., Dötsch C., Bug G., Ottmann O., Becker S., Pachl F. (2015). Mitotic arrest and slippage induced by pharmacological inhibition of Polo-like kinase 1. Mol. Oncol..

[243] Kreis N.-N., Sommer K., Sanhaji M., Krämer A., Matthess Y., Kaufmann M., Strebhardt K., Yuan J. (2009). Long-term downregulation of Polo-like kinase 1 increases the cyclin-dependent kinase inhibitor p21(WAF1/CIP1). Cell Cycle.

[244] Lénárt P., Petronczki M., Steegmaier M., Di Fiore B., Lipp J.J., Hoffmann M., Rettig W.J., Kraut N., Peters J.-M. (2007). The small-molecule inhibitor BI 2536 reveals novel insights into mitotic roles of polo-like kinase 1. Curr. Biol..

[245] Reindl W., Yuan J., Krämer A., Strebhardt K., Berg T. (2008). Inhibition of polo-like kinase 1 by blocking polo-box domain-dependent protein-protein interactions. Chem. Biol..

[246] Reindl W., Strebhardt K., Berg T. (2008). A high-throughput assay based on fluorescence polarization for inhibitors of the polo-box domain of polo-like kinase 1. Anal. Biochem..

[247] Scharow A., Raab M., Saxena K., Sreeramulu S., Kudlinzki D., Gande S., Dötsch C., Kurunci-Csacsko E., Klaeger S., Kuster B. (2015). Optimized Plk1 PBD Inhibitors Based on Poloxin Induce Mitotic Arrest and Apoptosis in Tumor Cells. ACS Chem. Biol..

[248] Yuan J., Sanhaji M., Krämer A., Reindl W., Hofmann M., Kreis N.-N., Zimmer B., Berg T., Strebhardt K. (2011). Polo-box domain inhibitor poloxin activates the spindle assembly checkpoint and inhibits tumor growth in vivo. Am. J. Pathol..

[249] Baxter M., Chapagai D., Craig S., Hurtado C., Varghese J., Nurmemmedov E., Wyatt M.D., McInnes C. (2020). Peptidomimetic Polo-Box-Targeted Inhibitors that Engage PLK1 in Tumor Cells and Are Selective against the PLK3 Tumor Suppressor. ChemMedChem.

[250] Matthess Y., Kappel S., Spänkuch B., Zimmer B., Kaufmann M., Strebhardt K. (2005). Conditional inhibition of cancer cell proliferation by tetracycline-responsive, H1 promoter-driven silencing of PLK1. Oncogene.

[251] Kolosenko I., Edsbäcker E., Björklund A.-C., Hamil A.S., Goroshchuk O., Grandér D., Dowdy S.F., Palm-Apergi C. (2017). RNAi prodrugs targeting Plk1 induce specific gene silencing in primary cells from pediatric T-acute lymphoblastic leukemia patients. J. Control. Release.

[252] Mason J.M., Wei X., Fletcher G.C., Kiarash R., Brokx R., Hodgson R., Beletskaya I., Bray M.R., Mak T.W. (2017). Functional characterization of CFI-402257, a potent and selective Mps1/TTK kinase inhibitor, for the treatment of cancer. Proc. Natl. Acad. Sci. USA.

[253] Lohse I., Mason J., Cao P.M., Pintilie M., Bray M., Hedley D.W. (2017). Activity of the novel polo-like kinase 4 inhibitor CFI-400945 in pancreatic cancer patient-derived xenografts. Oncotarget.

[254] Veitch Z.W., Cescon D.W., Denny T., Yonemoto L.-M., Fletcher G., Brokx R., Sampson P., Li S.-W., Pugh T.J., Bruce J. (2019). Safety and tolerability of CFI-400945, a first-in-class, selective PLK4 inhibitor in advanced solid tumours: A phase 1 dose-escalation trial. Br. J. Cancer.

[255] Lei Q., Xiong L., Xia Y., Feng Z., Gao T., Wei W., Song X., Ye T., Wang N., Peng C. (2018). YLT-11, a novel PLK4 inhibitor, inhibits human breast cancer growth via inducing maladjusted centriole duplication and mitotic defect. Cell Death Dis..

[256] Wong Y.L., Anzola J.V., Davis R.L., Yoon M., Motamedi A., Kroll A., Seo C.P., Hsia J.E., Kim S.K., Mitchell J.W. (2015). Cell biology. Reversible centriole depletion with an inhibitor of Polo-like kinase 4. Science.

[257] Suri A., Bailey A.W., Tavares M.T., Gunosewoyo H., Dyer C.P., Grupenmacher A.T., Piper D.R., Horton R.A., Tomita T., Kozikowski A.P. (2019). Evaluation of Protein Kinase Inhibitors with PLK4 Cross-Over Potential in a Pre-Clinical Model of Cancer. Int. J. Mol. Sci..

[258] Li F., Jo M., Curry T.E., Liu J. (2012). Hormonal induction of polo-like kinases (Plks) and impact of Plk2 on cell cycle progression in the rat ovary. PLoS ONE.

[259] Siegel R.L., Miller K.D., Fuchs H.E., Jemal A. (2021). Cancer Statistics, 2021. CA Cancer J. Clin..

[260] Kaku T., Ogawa S., Kawano Y., Ohishi Y., Kobayashi H., Hirakawa T., Nakano H. (2003). Histological classification of ovarian cancer. Med. Electron Microsc..

[261] Prat J. (2012). Ovarian carcinomas: Five distinct diseases with different origins, genetic alterations, and clinicopathological features. Virchows Arch..

[262] Kuhn E., Tisato V., Rimondi E., Secchiero P. (2015). Current Preclinical Models of Ovarian Cancer. J. Carcinog. Mutagen..

[263] Matulonis U.A., Sood A.K., Fallowfield L., Howitt B.E., Sehouli J., Karlan B.Y. (2016). Ovarian cancer. Nat. Rev. Dis. Primers.

[264] Cancer Genome Atlas Research Network (2011). Integrated genomic analyses of ovarian carcinoma. Nature.

[265] Yang D., Khan S., Sun Y., Hess K., Shmulevich I., Sood A.K., Zhang W. (2011). Association of BRCA1 and BRCA2 mutations with survival, chemotherapy sensitivity, and gene mutator phenotype in patients with ovarian cancer. JAMA.

[266] Milea A., George S.H.L., Matevski D., Jiang H., Madunic M., Berman H.K., Gauthier M.L., Gallie B., Shaw P.A. (2014). Retinoblastoma pathway deregulatory mechanisms determine clinical outcome in high-grade serous ovarian carcinoma. Mod. Pathol..

[267] Cheasley D., Wakefield M.J., Ryland G.L., Allan P.E., Alsop K., Amarasinghe K.C., Ananda S., Anglesio M.S., Au-Yeung G., Böhm M. (2019). The molecular origin and taxonomy of mucinous ovarian carcinoma. Nat. Commun..

[268] Verhaak R.G.W., Tamayo P., Yang J.-Y., Hubbard D., Zhang H., Creighton C.J., Fereday S., Lawrence M., Carter S.L., Mermel C.H. (2013). Prognostically relevant gene signatures of high-grade serous ovarian carcinoma. J. Clin. Investig..

[269] Murakami R., Matsumura N., Mandai M., Yoshihara K., Tanabe H., Nakai H., Yamanoi K., Abiko K., Yoshioka Y., Hamanishi J. (2016). Establishment of a Novel Histopathological Classification of High-Grade Serous Ovarian Carcinoma Correlated with Prognostically Distinct Gene Expression Subtypes. Am. J. Pathol..

[270] Zhang H., Zhang K., Xu Z., Chen Z., Wang Q., Wang C., Cui J. (2021). MicroRNA-545 suppresses progression of ovarian cancer through mediating PLK1 expression by a direct binding and an indirect regulation involving KDM4B-mediated demethylation. BMC Cancer.

[271] Zhang S., Jing Y., Zhang M., Zhang Z., Ma P., Peng H., Shi K., Gao W.-Q., Zhuang G. (2015). Stroma-associated master regulators of molecular subtypes predict patient prognosis in ovarian cancer. Sci. Rep..

[272] Ma S., Rong X., Gao F., Yang Y., Wei L. (2018). TPX2 promotes cell proliferation and migration via PLK1 in OC. Cancer Biomark..

[273] Raab M., Sanhaji M., Zhou S., Rödel F., El-Balat A., Becker S., Strebhardt K. (2019). Blocking Mitotic Exit of Ovarian Cancer Cells by Pharmaceutical Inhibition of the Anaphase-Promoting Complex Reduces Chromosomal Instability. Neoplasia.

[274] Noack S., Raab M., Matthess Y., Sanhaji M., Krämer A., Győrffy B., Kaderali L., El-Balat A., Becker S., Strebhardt K. (2018). Synthetic lethality in CCNE1-amplified high grade serous ovarian cancer through combined inhibition of Polo-like kinase 1 and microtubule dynamics. Oncotarget.

[275] Pujade-Lauraine E., Selle F., Weber B., Ray-Coquard I.-L., Vergote I., Sufliarsky J., Del Campo J.M., Lortholary A., Lesoin A., Follana P. (2016). Volasertib Versus Chemotherapy in Platinum-Resistant or -Refractory Ovarian Cancer: A Randomized Phase II Groupe des Investigateurs Nationaux pour l’Etude des Cancers de l’Ovaire Study. J. Clin. Oncol..

[276] Valsasina B., Beria I., Alli C., Alzani R., Avanzi N., Ballinari D., Cappella P., Caruso M., Casolaro A., Ciavolella A. (2012). NMS-P937, an orally available, specific small-molecule polo-like kinase 1 inhibitor with antitumor activity in solid and hematologic malignancies. Mol. Cancer Ther..

[277] Affatato R., Carrassa L., Chilà R., Lupi M., Restelli V., Damia G. (2020). Identification of PLK1 as a New Therapeutic Target in Mucinous Ovarian Carcinoma. Cancers.

[278] Fei H., Chen S., Xu C. (2020). Bioinformatics analysis of gene expression profile of serous ovarian carcinomas to screen key genes and pathways. J. Ovarian Res..

[279] Parrilla A., Barber M., Majem B., Castellví J., Morote J., Sánchez J.L., Pérez-Benavente A., Segura M.F., Gil-Moreno A., Santamaria A. (2020). Aurora Borealis (Bora), Which Promotes Plk1 Activation by Aurora A, Has an Oncogenic Role in Ovarian Cancer. Cancers.

[280] Deb B., Uddin A., Chakraborty S. (2018). miRNAs and ovarian cancer: An overview. J. Cell. Physiol..

[281] Chen S.-N., Chang R., Lin L.-T., Chern C.-U., Tsai H.-W., Wen Z.-H., Li Y.-H., Li C.-J., Tsui K.-H. (2019). MicroRNA in Ovarian Cancer: Biology, Pathogenesis, and Therapeutic Opportunities. Int. J. Environ. Res. Public Health.

[282] Jia X., Liu X., Li M., Zeng Y., Feng Z., Su X., Huang Y., Chen M., Yang X. (2018). Potential tumor suppressing role of microRNA-545 in epithelial ovarian cancer. Oncol. Lett..

[283] Syed N., Coley H.M., Sehouli J., Koensgen D., Mustea A., Szlosarek P., McNeish I., Blagden S.P., Schmid P., Lovell D.P. (2011). Polo-like kinase Plk2 is an epigenetic determinant of chemosensitivity and clinical outcomes in ovarian cancer. Cancer Res..

[284] Coley H.M., Hatzimichael E., Blagden S., McNeish I., Thompson A., Crook T., Syed N. (2012). Polo Like Kinase 2 Tumour Suppressor and cancer biomarker: New perspectives on drug sensitivity/resistance in ovarian cancer. Oncotarget.

[285] Ju W., Yoo B.C., Kim I.-J., Kim J.W., Kim S.C., Lee H.P. (2009). Identification of genes with differential expression in chemoresistant epithelial ovarian cancer using high-density oligonucleotide microarrays. Oncol. Res..

[286] Szenajch J., Szabelska-Beręsewicz A., Świercz A., Zyprych-Walczak J., Siatkowski I., Góralski M., Synowiec A., Handschuh L. (2020). Transcriptome Remodeling in Gradual Development of Inverse Resistance between Paclitaxel and Cisplatin in Ovarian Cancer Cells. Int. J. Mol. Sci..

[287] He Y., Wang H., Yan M., Yang X., Shen R., Ni X., Chen X., Yang P., Chen M., Lu X. (2018). High LIN28A and PLK4 co-expression is associated with poor prognosis in epithelial ovarian cancer. Mol. Med. Rep..

[288] Wang Z.J., Churchman M., Campbell I.G., Xu W.H., Yan Z.Y., McCluggage W.G., Foulkes W.D., Tomlinson I.P. (1999). Allele loss and mutation screen at the Peutz-Jeghers (LKB1) locus (19p13.3) in sporadic ovarian tumours. Br. J. Cancer.

[289] Macintyre G., Goranova T.E., de Silva D., Ennis D., Piskorz A.M., Eldridge M., Sie D., Lewsley L.-A., Hanif A., Wilson C. (2018). Copy number signatures and mutational processes in ovarian carcinoma. Nat. Genet..

[290] Kim A., Ueda Y., Naka T., Enomoto T. (2012). Therapeutic strategies in epithelial ovarian cancer. J. Exp. Clin. Cancer Res..

[291] Hennessy B.T., Coleman R.L., Markman M. (2009). Ovarian cancer. Lancet.

[292] Barton C.A., Hacker N.F., Clark S.J., O’Brien P.M. (2008). DNA methylation changes in ovarian cancer: Implications for early diagnosis, prognosis and treatment. Gynecol. Oncol..

[293] Reibenwein J., Krainer M. (2008). Targeting signaling pathways in ovarian cancer. Expert Opin. Ther. Targets.

[294] Smolle E., Taucher V., Pichler M., Petru E., Lax S., Haybaeck J. (2013). Targeting signaling pathways in epithelial ovarian cancer. Int. J. Mol. Sci..

[295] Sanchez-Vega F., Mina M., Armenia J., Chatila W.K., Luna A., La K.C., Dimitriadoy S., Liu D.L., Kantheti H.S., Saghafinia S. (2018). Oncogenic Signaling Pathways in The Cancer Genome Atlas. Cell.

[296] Li N., Zhan X. (2019). Signaling pathway network alterations in human ovarian cancers identified with quantitative mitochondrial proteomics. EPMA J..

[297] Cooke S.L., Ng C.K.Y., Melnyk N., Garcia M.J., Hardcastle T., Temple J., Langdon S., Huntsman D., Brenton J.D. (2010). Genomic analysis of genetic heterogeneity and evolution in high-grade serous ovarian carcinoma. Oncogene.

[298] Yamada H.Y., Gorbsky G.J. (2006). Spindle checkpoint function and cellular sensitivity to antimitotic drugs. Mol. Cancer Ther..

[299] Galimberti F., Thompson S.L., Ravi S., Compton D.A., Dmitrovsky E. (2011). Anaphase catastrophe is a target for cancer therapy. Clin. Cancer Res..

[300] Rieder C.L., Maiato H. (2004). Stuck in division or passing through: What happens when cells cannot satisfy the spindle assembly checkpoint. Dev. Cell.

[301] Zhang K., Kong X., Feng G., Xiang W., Chen L., Yang F., Cao C., Ding Y., Chen H., Chu M. (2018). Investigation of hypoxia networks in ovarian cancer via bioinformatics analysis. J. Ovarian Res..

[302] Mross K., Frost A., Steinbild S., Hedbom S., Rentschler J., Kaiser R., Rouyrre N., Trommeshauser D., Hoesl C.E., Munzert G. (2008). Phase I dose escalation and pharmacokinetic study of BI 2536, a novel Polo-like kinase 1 inhibitor, in patients with advanced solid tumors. J. Clin. Oncol..

[303] Arora S., Bisanz K.M., Peralta L.A., Basu G.D., Choudhary A., Tibes R., Azorsa D.O. (2010). RNAi screening of the kinome identifies modulators of cisplatin response in ovarian cancer cells. Gynecol. Oncol..

[304] Zhu Y., Liu Z., Qu Y., Zeng J., Yang M., Li X., Wang Z., Su J., Wang X., Yu L. (2020). YLZ-F5, a novel polo-like kinase 4 inhibitor, inhibits human ovarian cancer cell growth by inducing apoptosis and mitotic defects. Cancer Chemother. Pharmacol..

[305] Karakashev S., Zhang R.-G. (2021). Mouse models of epithelial ovarian cancer for preclinical studies. Zool. Res..

[306] Domcke S., Sinha R., Levine D.A., Sander C., Schultz N. (2013). Evaluating cell lines as tumour models by comparison of genomic profiles. Nat. Commun..

[307] Magnotti E., Marasco W.A. (2018). The latest animal models of ovarian cancer for novel drug discovery. Expert Opin. Drug Discov..

[308] Quail D.F., Joyce J.A. (2013). Microenvironmental regulation of tumor progression and metastasis. Nat. Med..

[309] Schwede M., Waldron L., Mok S.C., Wei W., Basunia A., Merritt M.A., Mitsiades C.S., Parmigiani G., Harrington D.P., Quackenbush J. (2020). The Impact of Stroma Admixture on Molecular Subtypes and Prognostic Gene Signatures in Serous Ovarian Cancer. Cancer Epidemiol. Biomark. Prev..

[310] Shaw T.J., Senterman M.K., Dawson K., Crane C.A., Vanderhyden B.C. (2004). Characterization of intraperitoneal, orthotopic, and metastatic xenograft models of human ovarian cancer. Mol. Ther..

[311] Fu X., Hoffman R.M. (1993). Human ovarian carcinoma metastatic models constructed in nude mice by orthotopic transplantation of histologically-intact patient specimens. Anticancer Res..

[312] Yi X.-F., Yuan S.-T., Lu L.-J., Ding J., Feng Y.-J. (2005). A clinically relevant orthotopic implantation nude mouse model of human epithelial ovarian cancer–based on consecutive observation. Int. J. Gynecol. Cancer.

[313] Zhang Y., Luo L., Zheng X., Yu T. (2016). An Advanced Orthotopic Ovarian Cancer Model in Mice for Therapeutic Trials. BioMed Res. Int..

[314] Cordero A.B., Kwon Y., Hua X., Godwin A.K. (2010). In vivo imaging and therapeutic treatments in an orthotopic mouse model of ovarian cancer. J. Vis. Exp..

[315] Yi C., Zhang L., Zhang F., Li L., Ling S., Wang X., Liu X., Liang W. (2014). Methodologies for the establishment of an orthotopic transplantation model of ovarian cancer in mice. Front. Med..

[316] Guo J., Cai J., Zhang Y., Zhu Y., Yang P., Wang Z. (2017). Establishment of two ovarian cancer orthotopic xenograft mouse models for in vivo imaging: A comparative study. Int. J. Oncol..

[317] Garcia Ribeiro R.S., Belderbos S., Danhier P., Gallo J., Manshian B.B., Gallez B., Bañobre M., de Cuyper M., Soenen S.J., Gsell W. (2019). Targeting tumor cells and neovascularization using RGD-functionalized magnetoliposomes. Int. J. Nanomed..

[318] Bochner F., Fellus-Alyagor L., Ketter D., Golani O., Biton I., Neeman M. (2020). Bimodal magnetic resonance and optical imaging of extracellular matrix remodelling by orthotopic ovarian tumours. Br. J. Cancer.

[319] Maniati E., Berlato C., Gopinathan G., Heath O., Kotantaki P., Lakhani A., McDermott J., Pegrum C., Delaine-Smith R.M., Pearce O.M.T. (2020). Mouse Ovarian Cancer Models Recapitulate the Human Tumor Microenvironment and Patient Response to Treatment. Cell Rep..

[320] Stuckelberger S., Drapkin R. (2018). Precious GEMMs: Emergence of faithful models for ovarian cancer research. J. Pathol..

[321] Zakarya R., Howell V.M., Colvin E.K. (2020). Modelling Epithelial Ovarian Cancer in Mice: Classical and Emerging Approaches. Int. J. Mol. Sci..

[322] Van der Horst P.H., van der Zee M., Heijmans-Antonissen C., Jia Y., DeMayo F.J., Lydon J.P., van Deurzen C.H.M., Ewing P.C., Burger C.W., Blok L.J. (2014). A mouse model for endometrioid ovarian cancer arising from the distal oviduct. Int. J. Cancer.

[323] Sherman-Baust C.A., Kuhn E., Valle B.L., Shih I.-M., Kurman R.J., Wang T.-L., Amano T., Ko M.S.H., Miyoshi I., Araki Y. (2014). A genetically engineered ovarian cancer mouse model based on fallopian tube transformation mimics human high-grade serous carcinoma development. J. Pathol..

[324] Zhai Y., Wu R., Kuick R., Sessine M., Schulman S., Green M., Fearon E., Cho K. (2017). High-grade serous carcinomas arise in the mouse oviduct via defects linked to the human disease. J. Pathol..

[325] Kim O., Park E.Y., Klinkebiel D.L., Pack S.D., Shin Y.-H., Abdullaev Z., Emerson R.E., Coffey D.M., Kwon S.Y., Creighton C.J. (2020). In vivo modeling of metastatic human high-grade serous ovarian cancer in mice. PLoS Genet..

[326] Perets R., Drapkin R. (2016). It’s Totally Tubular … Riding The New Wave of Ovarian Cancer Research. Cancer Res..

[327] Le Bras A. (2020). A new mouse model of ovarian cancer metastasis. Lab Anim..

[328] Roby K.F., Taylor C.C., Sweetwood J.P., Cheng Y., Pace J.L., Tawfik O., Persons D.L., Smith P.G., Terranova P.F. (2000). Development of a syngeneic mouse model for events related to ovarian cancer. Carcinogenesis.

[329] Walton J., Blagih J., Ennis D., Leung E., Dowson S., Farquharson M., Tookman L.A., Orange C., Athineos D., Mason S. (2016). CRISPR/Cas9-Mediated Trp53 and Brca2 Knockout to Generate Improved Murine Models of Ovarian High-Grade Serous Carcinoma. Cancer Res..

[330] Iyer S., Zhang S., Yucel S., Horn H., Smith S.G., Reinhardt F., Hoefsmit E., Assatova B., Casado J., Meinsohn M.-C. (2021). Genetically Defined Syngeneic Mouse Models of Ovarian Cancer as Tools for the Discovery of Combination Immunotherapy. Cancer Discov..

